# Effects of Different Physical Activity Approaches on Executive Functions in Primary School Children with ADHD: A Scoping Review with Methodological Reflections

**DOI:** 10.3390/bs16050703

**Published:** 2026-05-04

**Authors:** Gracia Cristina Villodres, Valentin Benzing, José Joaquín Muros

**Affiliations:** 1 Department of Didactics of Body Expression, Faculty of Education, University of Granada, 18071 Granada, Spain; jjmuros@ugr.es; 2 Institute of Sport Science, University of Bern, 3012 Bern, Switzerland; valentin.benzing@unibe.ch

**Keywords:** scoping review, physical activity, executive functions, primary schools, ADHD

## Abstract

Children with attention deficit/hyperactivity disorder (ADHD) often exhibit deficits in executive functions (EFs), which are crucial for self-regulation and academic performance. Physical activity (PA) has emerged as a promising non-pharmacological approach to support EF development in this population. This scoping review, guided by the PRISMA-ScR framework, examined PA interventions aimed at improving EFs in children aged 6–12 diagnosed with ADHD. Evidence was synthesized narratively by categorizing interventions according to PA modality, intensity, and the specific EF domains targeted, whilst describing the methodological characteristics of existing studies. A systematic search of PubMed, Cochrane Library, Web of Science, Scopus, and PsycINFO up to the end of 2024 yielded 55 peer-reviewed empirical studies involving 3863 participants. Both acute and chronic PA interventions were associated with improvements in EFs, with inhibitory control most commonly enhanced, followed by working memory and cognitive flexibility. Structured sports such as swimming and football, as well as multimodal interventions including physical–cognitive training and exergames, demonstrated positive effects. Intervention efficacy was influenced by factors such as intensity, duration, cognitive engagement, and context. However, variability in study designs, small sample sizes, and lack of standardization limited comparability. These findings suggest that PA interventions can support EF development in children with ADHD, particularly when activities are cognitively engaging. Future research should address methodological limitations and explore mediators and moderators in ecologically valid and theory-driven designs.

## 1. Introduction

Attention deficit hyperactivity disorder (ADHD) is one of the most prevalent neurodevelopmental disorders in childhood and adolescence, affecting between 2% and 12% of the pediatric population (Polanczyk et al., 2014; Rusca-Jordán & Cortez-Vergara, 2020). It is characterized by persistent symptoms of inattention, hyperactivity and impulsivity, which significantly interfere with the academic, family and social functioning of children who suffer from it. These symptoms are often accompanied by poor academic performance, self-regulation difficulties, and deficiencies in higher order cognitive skills, especially executive functions (EFs) (Arnold et al., 2020; Rubiales, 2014).

EFs are higher-order cognitive processes that enable individuals to regulate behavior, make decisions and adapt flexibly to changing demands. The core components of EFs, namely, inhibitory control, working memory and cognitive flexibility (Karr et al., 2018), are essential for learning, planning and academic achievement (Diamond, 2013; Munakata et al., 2012). In children with ADHD, EF deficits are commonly observed and have been associated with difficulties in sustaining attention, following instructions, regulating emotions and executing goal-directed behavior (Rubiales, 2014; Tamm et al., 2021). These impairments not only hinder school functioning but can also disrupt family dynamics, leading to increased stress among parents and caregivers (Rusca-Jordán & Cortez-Vergara, 2020).

Traditionally, ADHD treatment has been delivered through pharmacological approaches combined with psychosocial interventions (Cunill & Castells, 2020). While medications can mitigate the disorder’s core symptoms, their effectiveness is neither universal nor free of side effects, which has driven the exploration of complementary alternatives. Physical activity (PA) engagement has emerged as a promising non-pharmacological intervention to improve cognition and overall health during childhood.

From an educational psychology perspective, recent theoretical frameworks have provided deeper explanations for how PA can enhance executive and learning processes. The dual-process framework proposed by Zhang et al. (2025) posits that PA contributes to academic performance through both domain-general and domain-specific EFs, establishing a direct link between motor engagement and cognitive development. Complementarily, Zou et al. (2025) proposed the synergy of embodied cognition and cognitive load theory, suggesting that learning is optimized when bodily engagement supports cognitive processing and reduces extraneous load. Together, these perspectives offer a theoretical rationale for using physically and cognitively engaging activities to strengthen executive functioning and learning outcomes, particularly in educational contexts involving children with ADHD.

In this sense, numerous studies have shown that PA not only promotes physical well-being but can also enhance cognitive development, including attention, memory and executive processing, particularly in school-age children (Chaddock et al., 2010; Mavilidi et al., 2025). In particular, it has been highlighted that PA-based interventions with cognitive demands, such as active video games, may generate greater benefits than PA alone by combining physical effort with adapted cognitive challenges (Moreau & Conway, 2014; Cabral et al., 2025). Although this relationship has been less explored in children with disabilities, recent research suggests that PA engagement could have beneficial effects on EFs in children with ADHD, whilst also helping to reduce their symptoms and dependence on medication (Jeyanthi et al., 2019).

Despite growing interest, the literature on PA interventions for EF improvements in children with ADHD remains fragmented, with substantial variation in study design, intervention type, outcome measures used, and quality of evidence. Given the complex and heterogeneous nature of this field, a scoping review is an appropriate methodological approach to systematically map and describe existing evidence. Scoping reviews are particularly useful for clarifying key concepts, identifying research gaps, and informing future studies and practice when the literature is diverse and emerging (Tricco et al., 2018). Moreover, in consideration of the increased attention being placed on non-pharmacological approaches in educational and clinical contexts, it is critical to synthesize current findings in a way that is accessible and actionable for researchers, educators and healthcare professionals alike.

While previous reviews, such as Jeyanthi et al. (2019), have provided foundational insights, a new and updated scoping review is imperative. The landscape of educational and health research has evolved rapidly in recent years. The introduction of novel theoretical frameworks—such as the dual-process and embodied cognition models—provides new lenses through which to evaluate interventions. Furthermore, there is a growing need to synthesize recent post-pandemic evidence regarding non-pharmacological interventions in educational contexts. Thus, this review aims to bridge these recent theoretical advancements with the latest empirical outcomes.

The present study aims to explore the current state of evidence regarding the impact of PA interventions on the development of EFs in children aged 6–12 years diagnosed with ADHD. This review focuses on children aged 6–12 years, a critical developmental period when both ADHD symptoms typically emerge and EFs mature, with early EF abilities playing a key role in predicting lifelong outcomes such as academic achievement, health, wealth, and overall quality of life (Diamond, 2013). Moreover, limiting the age range to primary school years aligns with the intention of this scoping review to serve as a practical resource for primary school teachers, providing them with evidence-based ideas and strategies to inform classroom interventions for students with ADHD. Further, it places a specific focus on identifying effective strategies, common methodological features, and directions for future research and intervention design.

The research questions guiding this scoping review are as follows:

1. What types of PA interventions have been implemented to enhance EFs in children with ADHD?
2. Which components of EFs (inhibitory control, working memory and cognitive flexibility) have been most frequently targeted or reported as improved in studies on PA interventions?
3. What are the methodological features and outcomes of these studies in terms of design, sample characteristics, assessment tools and results?

## 2. Materials and Methods

### 2.1. Protocol and Register

Given the aforementioned context and in line with Preferred Reporting Items for Systematic Reviews and Meta-Analyses extension for Scoping Reviews (PRISMA-ScR) guidelines (Tricco et al., 2018) (see checklist in Supplementary Material S1), a scoping review was performed. Eligible studies were published between database inception and the 31 December 2024, with the last search being performed on the 1 February 2025. Included studies reported empirical analysis of the effectiveness of PA interventions on EFs in primary school students with ADHD. The review was registered with The Open Science Framework (OSF) (https://osf.io/945wd/overview, accessed on 24 June 2025).

### 2.2. Eligibility Criteria

Seven inclusion and seven exclusion criteria were employed for article selection.

Inclusion criteria were as follows: (1) intervention studies published in peer-reviewed journals in English or Spanish, (2) intervention studies conducted with 6–12-year-old school children, (3) PA included as the main intervention tool, (4) studies published between database inception and the 31st of December 2024, (5) studies examining the effect of PA engagement on EFs, (6) studies that consider ADHD as a unified construct rather than separate disorders or isolated symptoms, and (7) children with a formal diagnosis of ADHD or at high risk of ADHD (defined as presenting clinically significant symptoms assessed and identified via validated screening tools or standardized parent/teacher rating scales).

The exclusion criteria were as follows: (1) studies published in any language other than English or Spanish, (2) systematic reviews, meta-analyses, narrative reviews or other types of review studies, (3) inclusion of populations aged outside of the 6–12-year age range, (4) PA engagement not included as the main intervention tool, (5) studies not published between database inception and the 31 December 2024, (6) inclusion of high-performance athletes, (7) studies that only treat ADHD symptoms or subtypes as separate disorders rather than considering ADHD as a unified construct, and (8) individuals with alternative or additional diagnoses to ADHD that would potentially impact PA engagement, such as neurological or physical conditions (i.e., cerebral palsy, blindness, etc.).

### 2.3. Information Sources and Search Strategy

Studies included in the present systematic review were identified from the electronic databases PubMed, Cochrane Library, Web of Science, Scopus and PsycINFO. Descriptors were selected based on the specific thesauri attached to each database (medical subject headings [MeSH] and APA Thesaurus of Psychological Index). Search equations used in the databases included combinations of terms and descriptors in English organized into six domains (see Table 1). These terms were combined using the Boolean operators ‘AND’ and ‘OR’ to refine the search and were subsequently adapted for each database (see Supplementary Material S2). While the inclusion criteria permitted studies published in either English or Spanish, the search equations utilized only English descriptors due to the standard indexing practices of the selected international databases. Spanish-language articles were systematically captured and evaluated if they provided English-indexed titles, abstracts, or keywords.

### 2.4. Selection of Sources, Data Charting Process and Data Items

After identifying the initial pool of articles through database searches, all references were imported into two online platforms, Covidence Systematic Review Software (Veritas Health Innovation, Melbourne, Australia; web-based platform, accessed on 1 February 2025) and Rayyan Intelligent Systematic Review (Qatar Computing Research Institute, Doha, Qatar; web-based platform, accessed on 1 February 2025), to manage the screening and selection workflow (Kellermeyer et al., 2018). This process began with the automatic removal of duplicate records using Covidence (Babineau, 2014). To ensure completeness, a secondary manual check for duplicates was subsequently performed in Rayyan (Ouzzani et al., 2016).

Screening was conducted in two phases. First, titles and abstracts were evaluated using Rayyan based on the previously defined inclusion and exclusion criteria. Articles that passed this stage underwent a full-text review to assess their eligibility in more detail.

The screening process involved two independent reviewers (G.C.V. and J.J.M.. Any disagreements were resolved through discussion and, where necessary, following input from a third reviewer (V.B.). Unclear cases were debated collectively until consensus was reached.

Similarly to the screening phase, data extraction was conducted independently by two reviewers. Any discrepancies during this charting process were resolved through discussion and consensus or, if necessary, by consulting a third independent reviewer

To ensure consistency in data extraction, a structured checklist was used. This checklist contained standardized definitions and evaluation criteria, enabling both reviewers to assess studies from a common analytical framework. This checklist covered the following domains: (1) reference, (2) study aim (PA and EFs), (3) sample and age (range), (4) study design, (5) assessment method, (6) main findings (PA on EFs), (7) limitations and (8) conclusions. Further details on the checklist can be found in the Supplementary Material (available in the OSF preregistration, via https://osf.io/945wd/overview, accessed on 24 June 2025). To ensure consistency and transparency, the development of this checklist and the data charting process were guided by the holistic methodological framework of the PRISMA-ScR guidelines (Tricco et al., 2018), addressing the data extraction stages collectively.

## 3. Results

### 3.1. Selection of Sources of Evidence

Search equations were initially developed to filter studies based on titles and abstracts, with an additional restriction to include only studies published within the last 24 full calendar years (database inception to December 2024). A total of 2221 records were retrieved through database searches. These were distributed as follows: PubMed (*n* = 415), Cochrane Library (*n* = 46), Web of Science (*n* = 663), Scopus (*n* = 661) and PsycINFO (*n* = 436).

Prior to screening, duplicate removal was carried out in two stages. First, an automated process removed 920 duplicate records, resulting in 1301 references. A second manual check identified and removed 39 additional duplicates, leaving a total of 1262 documents for screening.

The titles and abstracts of identified records were then examined to remove studies that did not meet the inclusion criteria or that did meet any of the exclusion criteria. This led to the exclusion of 1105 records, leaving 157 reports for full-text review. However, eight of these could not be accessed. Despite being requested from the authors, they were not received within a 15-day period and so could not be included. This left 149 documents for eligibility assessment.

Following a detailed full-text evaluation, 94 reports were excluded for the following reasons: (1) conference abstracts or proceedings (*n* = 17), (2) study sample not aged between six and 12 years (*n* = 25), (3) participants diagnosed with additional neurological or physical conditions beyond ADHD that could impact PA engagement (*n* = 18), (4) study design used other than experimental or interventional (*n* = 23), (5) intervention did not involve PA (*n* = 3), (6) outcomes limited to symptom assessment, without cognitive measures (*n* = 5), and (7) studies that did not consider ADHD as a unitary construct (*n* = 3).

**Figure 1:**
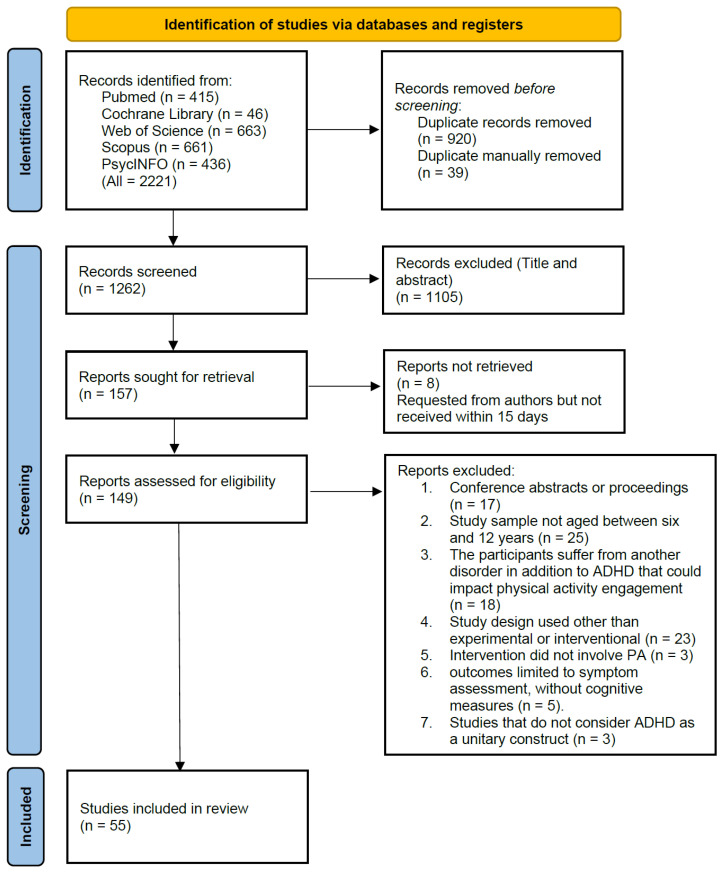
Flow diagram.

As a result, 55 studies met all eligibility criteria and were included in the final review. These studies are detailed in the data synthesis and summary sections of the manuscript. The full selection process is illustrated in Figure 1.

Although the relationship between PA and certain ADHD-related symptoms has been studied for decades, research prior to 2011 focused mainly on attention or hyperactivity in isolation, without addressing ADHD as a whole or its specific link to EFs. For this reason, the present scoping review includes only studies published from 2011 onwards, as this is when research began to consider ADHD as an integrated construct and more specifically examined the impact of PA engagement on EFs in this population.

### 3.2. Characteristics of Sources of Evidence

The 55 included interventions involved a total of 3863 children with a mean age of between six and 12 years. The sample comprised a significantly higher proportion of boys than girls. This gender imbalance is consistent with epidemiological evidence indicating that ADHD is more frequently diagnosed in boys than in girls, particularly during childhood (Centers for Disease Control and Prevention, 2024).

In terms of the geographical distribution of studies, research was conducted in a range of countries, with the United States presenting the backdrop to the highest proportion of studies (25.49%). This was followed by Taiwan (19.61%), Iran (17.65%), Spain (7.84%), Switzerland (7.84%) and China (7.84%). Other countries, represented to a lesser extent, include South Korea (5.88%), Tunisia (3.92%), Canada (3.92%), and individual contributions (1.96%) from Germany, Scotland, Israel, Mexico, Hungary, Brazil and Hong Kong.

Included studies are based on interventions that develop different types and intensities of PA and exercise (aerobic exercise, acute aerobic exercise, high-intensity exercise, exercise with cognitive training, exercise with relaxation, active video games or exergames, sports structured, classroom exercise, etc.), and study the effect of this on the main EFs such as inhibitory control, working memory and cognitive flexibility, as well as attention and self-regulation. For the assessment of EFs, computerized tasks are generally used (i.e., Go–No-Go Task, Stroop Test, Flanker, Wisconsin Card Sorting Test [WCST], Digit Span, Corsi Block Tapping Test, N-Black Test, Visual Pursuit Test, Behavior Rating Inventory of Executive Function [BRIEF], and the Tower of London Test) and, in some cases, electroencephalography (EEG) is used.

Table S1 (Supplementary Material S3) presents more detailed information on the included studies, including citation, study aim, sample and age range, study design, assessment method, main statistical findings regarding the effects of PA engagement on EFs, limitations, and conclusions.

### 3.3. Characteristics of Intervention Studies

“Any type of PA can provide benefits in children with ADHD” (Ziereis & Jansen, 2015). But are all types of PA designed and implemented in the same way? Next, a closer look at the literature reveals nuanced differences depending on how interventions are structured and delivered.

#### 3.3.1. Acute PA Interventions

Acute PA interventions, typically involving short, one-time exercise sessions, have been used to explore their immediate effects on EFs in children with ADHD. The evidence is presented in two parts: first, general short-term interventions, and second, studies focused specifically on acute aerobic exercise.

##### Short-Term Interventions Based on PA and EFs

Gawrilow et al. (2016) and, more recently, Miklós et al. (2020) conducted short-term interventions and found positive effects of PA engagement on EFs. In this regard, Gawrilow et al. (2016) randomly assigned 47 children with ADHD to either a PA condition in which they jumped on a trampoline for five minutes, or a sedentary control condition in which they engaged in coloring in. Findings indicated that children who engaged in PA showed significant improvements in response inhibition and made fewer errors in a cognitive task compared to the sedentary group. Similarly, Miklós et al. (2020) examined the effects of a single 20 min session of moderate-intensity PA on attention and EFs in 150 children, including 50 with unmedicated ADHD, 50 with medicated ADHD, and 50 typically developing controls. Participants were randomly assigned to either a PA condition (cycling while watching a cartoon) or a sedentary control condition (watching a cartoon whilst sitting). The PA intervention significantly improved performance on two out of 15 measures when compared to the CG (number of total errors and errors with a distractor in the distractibility task), although it was found to be less effective over time for unmedicated children with ADHD.

Nevertheless, recently, Barudin-Carreiro et al. (2024) conducted a randomized pilot trial to examine the feasibility and preliminary effects of low-intensity PA on EFs in 22 children with ADHD. Participants were assigned to one of three 20 min conditions: walking, standing, or sitting, whilst listening to music. The sitting group showed the greatest improvement in inhibition, while the standing group showed the largest gains in cognitive flexibility and problem-solving.

##### Acute Aerobic Exercise and EFs

As noted above, there is evidence that PA engagement has the potential to enhance EFs in children with ADHD. In this regard, various experimental studies have demonstrated that engaging in exercise, specifically aerobic exercise, can lead to improvements in the EFs of children with ADHD.

Several studies have examined the effects of single-session aerobic exercise, also referred to as acute aerobic exercise, on EFs. For instance, Y. K. Chang et al. (2012) investigated the effects of a single session of moderate-intensity aerobic exercise on EFs in 40 children with ADHD. Participants were randomly assigned to an EG that completed 30 min of treadmill exercise (including warm-up and cool-down) or to a CG that watched a video. The outcomes showed significant improvements in inhibition and cognitive flexibility in the exercise group.

Similarly, Yu et al. (2020) examined the effects of acute moderate-intensity aerobic exercise on inhibitory control in children with ADHD. The study involved 30 children who participated in two sessions, with one consisting of 30 min of exercise and another comprising a control session in which participants watched a video. Findings indicated that the group engaging in moderate-intensity acute aerobic exercise demonstrated greater accuracy on inhibitory control tasks (90.4% accuracy compared to 83.7% in the CG). Additionally, exercise enhanced brain activity related to attention and control. This helps children identify conflicting information faster and respond more effectively.

Furthermore, Chuang et al. (2015) studied the effects of acute aerobic exercise on motor inhibition and attentional preparation in 19 children with ADHD using a randomized crossover design. Each child completed a 30 min treadmill aerobic exercise session and a control session in which they watched a video on separate days. After each condition, participants performed a task that measured their ability to pay attention and control their impulses. Outcomes showed that, after exercising, participants reacted more quickly (441.6 ms vs. 470.86 ms), without making more mistakes. Exercise also changed brain activity related to attention and readiness to respond in a way that makes it more balanced in the face of different types of stimuli.

In a similar sense, Piepmeier et al. (2015) focused on the impact of acute exercise on familiar and specific cognitive tasks by examining the effects of a single 30 min session of moderate-intensity cycling exercise on EF performance in 32 children, of which 14 were diagnosed with ADHD and 18 were not. Through a randomized within-subject crossover design, participants completed both an exercise condition (5 min warm-up, 20 min moderate cycling, and 5 min cool-down) and a control condition involving watching a nature documentary. Findings revealed that exercise significantly improved performance on the Stroop Task, via enhanced inhibitory control and processing speed, with comparable benefits for children both with and without ADHD. No significant effects were observed for planning or cognitive flexibility.

Likewise, continuing with the focus on familiar and specific cognitive tasks, Ludyga et al. (2017) examined the acute effects of aerobic and coordinative exercise on inhibitory control and attentional resource allocation in 36 children (18 with ADHD and 18 typically developing children). Participants completed a Flanker task before and after each of the three conditions. The three conditions were: (1) 20 min of moderate aerobic exercise (cycling at 65–70% HRmax), (2) 20 min of coordinative exercise (object control and bilateral coordination), and (3) a 20 min control condition (watching a video). Both exercise types improved performance in the ADHD group, with significantly reduced reaction times and increased P300 amplitude (an index of attentional allocation). Aerobic exercise elicited larger improvements than coordinative exercise in the ADHD group. In contrast, no significant differences between exercise conditions were observed in the non-ADHD group.

Finally, a study conducted by Tsai et al. (2021) further reinforces these findings. This study examined the way in which the intensity of acute aerobic exercise affects inhibitory control in 25 children with ADHD. Sessions lasted 30 min and comprised a 5 min warm-up, 20 min of treadmill running at different intensities (low, moderate and vigorous) and a 5 min cool-down. After each session, participants completed a Flanker task to evaluate inhibitory control and attention. Outcomes indicated that, relative to high-intensity exercise, low- and moderate-intensity exercise led to better inhibitory control, as evidenced through children’s shorter reaction times on the Flanker task.

#### 3.3.2. Chronic PA Interventions

Chronic PA interventions, involving repeated sessions over several weeks, aim to assess the long-term effects of exercise on EFs in children with ADHD. The following evidence is grouped into general long-term programs and interventions based specifically on aerobic exercise.

##### Long-Term Interventions Based on PA and EFs

Kang et al. (2011) and, more recently, Liang (2023) implemented longer-term interventions. In this context, Kang et al. (2011) conducted a six-week intervention involving 90 min sessions twice a week, incorporating aerobic, coordination and jumping exercises. The study assessed the effects of sports therapy on attention symptoms and EFs in 28 children with ADHD. After six weeks, the sports therapy group showed significant improvements in EF measures such as attention, processing speed and attentional control compared to an educational CG. Likewise, Liang (2023) explored the effects of PA engagement on EFs in children with ADHD. The study involved 120 participants (40 in a PA EG, 40 who acted as a waitlist control group [CG], and 40 typically developing children). The 12-week intervention comprised 36 sessions of combined moderate-intensity (60–80% HRmax) aerobic and cognitive-engaging exercises, with each session lasting 60 min (10 min warm-up, 20 min of aerobic activity, 20 min of cognitive activity, and 10 min cool-down). The findings demonstrated significant improvements in inhibitory control, working memory and cognitive flexibility in the PA group, with effects being sustained 12 weeks post-intervention.

Conversely, some studies report less conclusive outcomes. Fedewa et al. (2021) examined the effects of a 16-week structured PA intervention (30 min per session, three sessions per week) involving 59 children. While no significant improvements were found in executive functioning or ADHD symptoms according to parent and teacher reports, exploratory analyses suggested that activity intensity may play a moderating role. Specifically, time spent in vigorous or light PA was associated with better EFs, while moderate-intensity activity was unexpectedly linked to poorer EF outcomes based on teacher reports. These findings underscore the potential relevance of PA intensity when designing interventions for children with ADHD.

##### Long-Term Aerobic Exercise and EFs

Long-term aerobic exercise programs have shown sustained benefits on EFs in children with ADHD. Evidence supporting this has been provided by Suazo et al. (2019), Verret et al. (2012) and Mohammadi-Orangi et al. (2021).

The study conducted by Suazo et al. (2019) evaluated the impact of a PA program on attention in children with ADHD. The sample included 24 children (13 in the EG and 11 in the CG). The intervention consisted of a six-week aerobic exercise program with two one-hour sessions per week that comprised a warm-up, 30 min of moderate-to-high intensity aerobic games, and a cool-down. Outcomes indicated significant improvements in attention quality and sustained attention in the intervention group, although no improvements in impulsivity were observed.

In a similar vein, Verret et al. (2012) conducted a randomized controlled trial with 21 Canadian children. Those in the EG (*n* = 10) participated in a 10-week PA program that comprised three 45 min sessions per week. The program included aerobic, resistance and motor skill exercises, all of which were performed at moderate-to-high intensity and were monitored via heart rate. Outcomes revealed significant improvements in information processing speed and sustained auditory attention compared to the control.

Similarly, a study conducted by Mohammadi-Orangi et al. (2021) investigated the effects of an aerobic exercise program combined with music on the cognitive abilities of working memory, perceptual reasoning and processing speed in 36 children with ADHD who were equally distributed between experimental and control groups. The EG engaged in an eight-week program that comprised three 60 min sessions per week and were delivered in a nonlinear pedagogical setting. In contrast, the CG continued with traditional school activities. Outcomes demonstrated significant improvements in the EG. Specifically, working memory, perceptual reasoning and processing speed increased by 6.50, 6.23 and 8.34 units, respectively, whereas the CG exhibited improvements of less than one unit for each aspect.

Furthermore, the benefits of sustained aerobic exercise have also been observed at a neurophysiological level. Jiang et al. (2022) examined the effects of an eight-week moderate-intensity aerobic exercise program (jump rope, three times per week for 30 min) in 17 children with ADHD, using cognitive tasks and functional magnetic resonance imaging (fMRI). Outcomes revealed significant improvements in inhibitory control, with faster reaction times on the Flanker task after the intervention. At the brain level, increases in regional homogeneity (ReHo) were observed in the left middle frontal gyrus and right superior frontal gyrus, as well as increased degree centrality (DC) in the right posterior cingulate cortex (regions associated with executive control).

#### 3.3.3. Intensity in PA

Despite findings reported previously by Tsai et al. (2021), which showed better inhibitory control after low- and moderate-intensity exercise, other studies have reported positive outcomes following high-intensity exercise on the EFs of children with ADHD.

##### High-Intensity Exercise and EFs

For instance, a study conducted by Suárez-Manzano et al. (2021) and Suárez-Manzano et al. (2022) assessed the effects of a cooperative high-intensity interval training program (C-HIIT) on inhibitory control in children with ADHD. The study involved 52 children who were divided into a C-HIIT intervention group (*n* = 24) and a non-intervention CG (*n* = 28). The program lasted 10 weeks and consisted of 30 min sessions twice a week that comprised a warm-up, C-HIIT exercises and a cool-down. Outcomes indicated that the C-HIIT group experienced significant improvements in inhibitory control and selective attention, while the CG exhibited no significant changes. The authors concluded that supervised C-HIIT is effective for enhancing inhibitory control in children with ADHD.

Additionally, a recent study conducted by Sun et al. (2024) examined the effectiveness of a game-based high-intensity interval training program (GameHIIT) compared to a game-based structured aerobic exercise program (GameSAE) and a CG. The study recruited a sample of 63 children with ADHD. Participants were randomly assigned to one of three groups: GameHIIT, GameSAE, or a no-treatment CG. The intervention lasted eight weeks, with 30 min sessions twice a week for the GameHIIT group and approximately 1 h sessions for the GameSAE group. During this period, the CG maintained their regular levels of PA. In this case, no significant effects on EFs were found in comparison to the CG, with the exception of a notable improvement in self-monitoring in the GameSAE group.

#### 3.3.4. Structured PA and EFs

Continuing with the review of the existing literature on the effects of PA on EFs in children with ADHD, some studies have reported positive impacts on EFs in the present population following targeted and structured PA programs, or specific sports, with aquatic activities especially standing out in this regard.

##### Specific Sports Interventions

For example, Y. K. Chang et al. (2014) analyzed the impact of an eight-week aquatic exercise program on inhibitory control in 27 children with ADHD. Participants were divided into an aquatic exercise group (14 participants) and a CG (13 participants). The intervention consisted of two weekly 90 min sessions including aerobic and coordinative exercises. Outcomes revealed that the aquatic exercise group experienced significant improvements in inhibitory control (from 88.64% to 94.31%).

Similarly, C. J. Huang et al. (2017) conducted an aquatic intervention with 32 children with ADHD to investigate the effects of aerobic exercise on resting-state EEG. The intervention consisted of an eight-week aquatic aerobic program comprising two 90 min sessions per week, during which 40% of available time was devoted to aerobic exercise. Meanwhile, the CG refrained from exercise. Findings revealed that the exercise group presented significantly lower theta/alpha ratios and greater alpha power in the frontal and central brain regions compared to the CG. This suggests that aerobic exercise may reduce cognitive deficits associated with ADHD by improving attentional control.

Continuing with aquatic exercises, specifically with the sport of swimming, a study conducted by Hattabi et al. (2019) investigated the effect of a recreational swimming program on cognitive functions in 40 children with ADHD. Participants were randomly assigned to an EG or a CG. The intervention program consisted of 36 swimming sessions, which were held three times per week over 12 weeks and comprised a 5 min warm-up, 80 min of moderate-intensity aquatic aerobic exercises, and a 5 min cool-down. Findings highlighted significant improvements in memory accuracy, selective attention and inhibitory processes in the swimming group compared to the CG.

Similarly, a study conducted by Silva et al. (2020) investigated the effects of a swimming program on cognition in children with ADHD. The sample included 20 children diagnosed with ADHD who were randomly assigned to a training group that participated in an eight-week swimming program (two sessions per week) or a no-exercise CG. The program consisted of 45 min sessions comprising a warm-up, swimming exercises, recreational activities and a cool-down. Outcomes regarding EFs indicated a significant increase in cognitive flexibility and selective attention in the EG compared to the CG.

This type of intervention has continued to be implemented in more recent research. For example, a study conducted by Hattabi et al. (2022) evaluated the impact on EFs of a 12-week recreational swimming program as an alternative treatment for 40 children with ADHD. The swimming program consisted of three 90 min sessions per week that were supervised by professional coaches and comprised a 15 min warm-up, 70 min of aquatic exercises, and a 5 min cool-down. Findings demonstrated significant improvements in inhibitory control and academic performance in the EG compared to the CG.

##### Structured Exercise Programs

In addition to swimming and other aquatic interventions, other studies have investigated the effects on EFs of different sports, such as football (soccer). A study conducted by Jun (2023) examined the effects of football on the EFs of children diagnosed with ADHD. Participants were randomly assigned to an EG and a CG. The EG participated in a six-week football intervention, in addition to their school’s regular sports activities, while the CG only took part in standard physical education classes. Outcomes showed that the football intervention significantly improved inhibitory control and cognitive flexibility compared to both the CG and the conventional physical education group. However, no significant differences were observed in working memory between the football group and the conventional physical education group.

In addition to football, other sports such as martial arts have also been investigated regarding their impact on EFs in children with ADHD. For instance, Ludyga et al. (2023) evaluated the effects of judo training on behavioral and neurocognitive inhibition in children with ADHD and children born preterm. The intervention consisted of 60 min judo sessions that were held twice a week for 12 weeks. Findings indicated improvements in inhibition among children born preterm, although no significant effects were observed in the ADHD group. This suggests that benefits may vary depending on the population under study and the specific cognitive demands of the sport.

In addition to specific sports, other researchers have focused on studying structured exercise programs. For example, Memarmoghaddam et al. (2016) examined the effectiveness of an exercise program on EF in 40 boys with ADHD aged between seven and 11, who were randomly assigned to an EG or a CG. The program consisted of 24 90 min sessions delivered over eight weeks, which comprised warm-up exercises, aerobic activities, directed tasks, station-based exercises and ball games. Findings revealed that the program had a significant effect on cognitive and behavioral inhibition, with notable improvements in reaction time and accuracy.

In terms of more recent studies on the topic, a study conducted by Z. Huang et al. (2024) also stands out. This study examined the effects of an eight-week rope skipping exercise (RSE) program on working memory in 55 children with ADHD and 27 typically developing children. Participants were randomly assigned to one of three groups: (1) an AWRSE group (children with ADHD who performed RSE), (2) an AWSG group (children with ADHD who participated in sports-based games), and (3) a CG (typically developing children). The AWRSE group completed 60 min RSE sessions twice a week, while the other two groups participated in similar sport-based games. Outcomes demonstrated that the AWRSE group experienced significant improvements in working memory after the eight-week intervention. In contrast, the other groups did not show significant changes in this domain.

##### Mind–Body and Relaxation Practices

Relaxation techniques embedded within exercise have also been examined on multiple occasions. Chou and Huang (2017) evaluated the effects of an eight-week yoga program on sustained attention and discrimination function in 49 children with ADHD. Twenty-four children were assigned to a yoga group and 25 to a CG. The yoga program included 40 min sessions twice a week that consisted of a 10 min warm-up, 20 min of yoga activities focused on concentration, balance, and attention, and a 10 min cool-down. Outcomes revealed that the yoga group significantly improved in both accuracy and reaction time in comparison with the CG, which indicated enhanced sustained attention and discrimination ability. Similarly, Rezaei et al. (2018) also examined the effect of yoga on memory and cognition in children with ADHD. A total of 21 elementary school students with ADHD were randomly assigned to neurofeedback, yoga, or control. Both interventions consisted of 24 45 min sessions delivered over eight weeks. The yoga group, in particular, showed significant improvements in terms of response errors and memory.

In this context, mindfulness-based interventions have also been explored. Bigelow et al. (2021) examined the effects of mindfulness meditation and acute exercise on EFs in 16 children with ADHD. Participants completed three 10 min interventions (moderate-intensity cycling, guided mindfulness meditation via a mobile application, and a reading condition as a control) in a randomized order. Findings indicated that mindfulness meditation produced significant improvements in inhibitory control, working memory and task switching, both immediately and in delayed assessments, whereas acute exercise did not produce changes in these areas.

In line with this, Nejatifar et al. (2022) evaluated the impact of Dohsa-Hou rehabilitation on response inhibition and sustained attention in 30 boys with ADHD using a quasi-experimental randomized pre-test–post-test design. The EG (*n* = 15) underwent an eight-month rehabilitation program based on breathing techniques, body movements, and tasks designed to enhance attention mechanisms and self-control. This program was delivered in monthly one-hour sessions with eight sessions in total. The CG (*n* = 15) did not receive any intervention. Outcomes showed significant improvements in the EG relative to the CG in terms of response inhibition and sustained attention.

#### 3.3.5. Educational Context

Given the amount of time children spend in school, the educational setting has become a key context for implementing PA interventions aimed at enhancing EFs. Some studies have explored how structured PA sessions, integrated within or around academic tasks, can influence cognitive and academic outcomes in students with ADHD.

##### Classroom Exercise and EFs

In terms of more traditional approaches, such as aerobic exercise-based interventions, Pontifex et al. (2013) investigated the impact of a single session of moderate aerobic exercise on neurocognition and academic performance in 40 children (20 with ADHD and 20 healthy controls). Participants completed three sessions in which 20 min of treadmill exercise was alternated with 20 min of reading. Findings revealed that both groups improved their response accuracy to a greater extent after exercise (87.1% vs. 83.5% after reading). Among the ADHD group, a significant increase in post-error slowing was observed, which suggests enhanced action monitoring. Additionally, improvements were found in reading and arithmetic skills.

With regard to brief exercise interventions in this setting, Hill et al. (2011) studied the impact of a short-duration exercise program (10–15 min daily) on EFs in primary school children. For two weeks, children engaged in 10–15 min of teacher-led moderate PA each day, followed by a control week without exercise. Findings revealed significant improvements in working memory, sustained attention and cognitive flexibility by the end of the second week of exercise across all participants. This suggests that PA engagement can enhance cognitive performance in children with and without ADHD symptoms. Similarly, Smith et al. (2013) examined the effect of a before-school PA intervention on EFs in children with ADHD symptoms. The intervention consisted of 30 min of moderate-to-vigorous PA, which was delivered through playful motor tasks, conducted over eight weeks. Changes in several areas, including EFs, were assessed using standardized tests and behavioral observation. Findings demonstrated significant improvements in EFs, especially in response inhibition.

In contrast, de la Merced-García and Jarauta (2022) evaluated the impact of adopting a more innovative and long-term approach, via classroom bike desks, on academic performance and attention in students with ADHD. The study involved 13 participants who were assigned to the EG with bike desks or a control. The intervention lasted 14 weeks. Academic performance and attention were assessed using validated tests, as well as teacher and parent observations and reports. Nonetheless, in this case, no significant effects were found.

#### 3.3.6. Combined or Multimodal Interventions

##### Exercise Combined with Cognitive Training and EFs

Some studies assert that, in light of the challenge of transferring the benefits of exercise to EFs, in order to be truly effective, interventions should combine physical exercise with specific cognitive training.

In this sense, interventions combining general physical exercise with specific cognitive tasks have produced positive outcomes. For example, Lee et al. (2017) examined the impact of a combined exercise program on frontal EFs and brain activity in children with ADHD using EEG. This pilot study involved 18 children with ADHD who were randomly assigned to an exercise group or a CG. The program consisted of 60 min sessions of rope jumping and ball exercises. Sessions were performed three times a week for 12 weeks and progressed in intensity from 45% to 75% of heart rate reserve. Outcomes indicated a significant increase in β-waves in EEG tasks for the exercise group, which were associated with improved attention and focused thinking.

Similarly, Wexler et al. (2021) investigated an integrated cognitive training program that combined computer-based exercises and PA in 93 children with ADHD. Participants were randomly assigned to a 15-week cognitive training group or a usual treatment CG. The intervention comprised cognitive exercises that targeting focused attention, response inhibition and working memory, as well as physical exercises designed to reinforce EFs, together with performance of a group game called the Good Behavior Game. At the end of the intervention period, the intervention group reported significant improvements in working memory, attention and inhibition.

More recently, Nejati and Derakhshan (2021) reinforced the aforementioned findings by examining the effects of combining cognitive tasks with a PA program on EFs and behavioral traits in 30 children with ADHD. Participants were randomly assigned to one of two groups. The first received a cognitive enhancement and rehabilitation exercise (EXCIR) program, which combined physical exercise with cognitive tasks, while the second, an active CG, performed only aerobic exercise (running). Both interventions consisted of 10–12 sessions lasting 40–50 min conducted three times a week over a period of four to five weeks. Outcomes revealed significant improvements in working memory, cognitive flexibility and inhibitory control in the EXCIR group. These improvements were statistically significant and sustained one month after the intervention ended.

On the other hand, specific programs employing innovative tools to integrate physical exercise and cognitive demands have also yielded promising results. For example, a study conducted by Nejati (2021) evaluated the effectiveness of the Balance-based Attentive Rehabilitation of Attention Networks (BARAN) program in 29 children with ADHD. This program combines balance training with cognitive tasks that are delivered in 12–15 sessions lasting 40 to 50 min. Sessions were delivered three times per week over a period of four to five weeks. Outcomes demonstrated significant improvements in the group that received the BARAN intervention, particularly in working memory, where accuracy improved by 14.8% and response time decreased significantly. Further, whilst cognitive flexibility improved in both groups, those in the BARAN group exhibited greater reductions in perseverative errors and response times. With regard to inhibitory control, improvements were only observed in the BARAN group.

Similarly, Liang et al. (2022) evaluated the effects of a combined aerobic and neurocognitive exercise intervention on EFs in 120 children, of which 40 were healthy children, 40 had ADHD and 40 had ADHD on a waitlist. Participants were randomly assigned to the EG or to a waitlist CG. The intervention lasted 12 weeks and consisted of 36 60 min sessions that were held three times per week and comprised a warm-up, aerobic exercises, neurocognitive exercises, and a cool-down. Outcomes indicated significant improvements in all EF measures in the EG compared to the CG.

Additionally, some interventions have focused on specific sports with inherent physical and cognitive components. Pan et al. (2019) examined the effects of a table tennis (including basic skill training and EF-related exercises) intervention on EFs in 60 boys with ADHD. Participants were divided into three groups: (1) a training group comprising 15 children with ADHD, (2) a non-training group comprising 15 children with ADHD, and (3) a control group consisting of 30 typically developing children. Intervention sessions were held twice per week, with sessions lasting 70 min each. Outcomes showed that, in addition to improvements in locomotor and object control motor skills in the ADHD training group compared to the non-training groups, significant improvements also emerged in terms of inhibitory control and overall performance.

Finally, studies that incorporated digital platforms also highlight the value of combined interventions. For instance, Shams et al. (2021) examined the impact of cognitive rehabilitation combined with physical exercise on sustained, selective and alternating attention in 40 girls with ADHD. The main aim of this was to assess the effect of this combination of interventions on different types of attention. Participants were randomly assigned to one of four groups: (1) cognitive rehabilitation, (2) physical exercise, (3) a combination of both interventions, and (4) a CG. Cognitive rehabilitation was delivered through the My Brain application in 16 60 min sessions. Findings indicated that the combined group (cognitive rehabilitation + physical exercise) performed significantly better in terms of sustained, selective, and alternating attention compared to the CG, committing fewer errors and achieving higher scores on all attention tests when compared with the other groups.

##### Exergames (Active Video Games) and EFs

In recent years, the use of exergames (active video games) has emerged as an innovative strategy for improving EFs in children with ADHD through the integration of technology, PA and cognitive stimulation. Several studies have now explored efficacy and feasibility in this context.

For example, Garcia-Lara et al. (2016) evaluated an intervention program that employed the Play Attention Interface Box software (Play Attention Inc., Asheville, NC, USA; model PA H004) in an intervention with 19 children with ADHD. Seven participants were assigned to the intervention group and 12 to the CG. The intervention, which lasted for approximately four months, combined brain activity monitoring and cognitive exercises through self-instruction sessions and software use. Outcomes highlighted significant improvements in attention and visual memory in the intervention group, while the CG exhibited only slight improvements.

Similarly, Weerdmeester et al. (2016) investigated the effectiveness and feasibility of the active video game, Dragon, in 73 children aged six to 13 years with ADHD. Children were randomly assigned to an EG, which played Dragon, or to a CG, which played Angry Birds. The intervention consisted of six 15 min sessions held over three weeks and totaling 1.5 h of gameplay. Although outcomes were encouraging in that the intervention achieved a significant reduction in impulsivity, both groups performed worse on the Go–No-Go task (which assesses inhibitory control) at the end of the study.

Furthermore, Benzing and Schmidt (2017) investigated the effects of a cognitively and physically demanding exergame on the EFs of 66 children with ADHD. Participants were assigned to an intervention group, which completed 30 min exergaming sessions three times per week over eight weeks, or to a waitlist CG. The game used, Shape UP (Xbox Kinect), involves tasks that demand both physical and cognitive effort. Outcomes indicated significant improvements in the intervention group in EFs through improved inhibition, working memory and cognitive flexibility.

In a similar vein, Benzing and Schmidt (2019) employed the same video game in a randomized controlled trial with 51 children with ADHD. Following intervention, significant improvements were found in inhibition and cognitive flexibility, although not in working memory updating. These improvements were achieved after eight weeks of intervention and were also associated with higher levels of enjoyment and engagement during sessions.

Additionally, a previous study conducted by Benzing et al. (2018) evaluated the acute effects of a single 15 min session of exergaming (Shape UP) in 46 children with ADHD. Compared to a group that watched a documentary, the exergaming group exhibited significantly faster reaction times during tasks that assessed inhibition and task switching, although no differences were found in accuracy or visual working memory. This finding suggests that even a brief session of cognitively demanding PA may have immediate effects on specific components of executive functioning.

Shema-Shiratzky et al. (2019) evaluated the feasibility and efficacy of conducting a motor cognition training program using virtual reality with 14 children with ADHD. The program consisted of 18 training sessions held over six weeks, during which participants walked on a treadmill while avoiding virtual obstacles. The purpose of this was to promote dual-task training. Participants showed significant improvements, not only in social problems, gait step regularity, and psychosomatic behavior after training, but also in memory. Effects were maintained for six weeks post-intervention.

Additionally, other studies have compared interventions involving traditional PA with those using exergames. For example, S. H. Chang et al. (2022) investigated the impact of table tennis training (both traditional and simulated) on EFs in 48 children with ADHD. Participants were randomly assigned to one of three groups: (1) real table tennis training, (2) a simulated table tennis game (exergame), and (3) a CG receiving no additional training. Over a 12-week period, children in the intervention groups attended three one-hour sessions per week, totaling 36 sessions. Findings showed that both the real and simulated table tennis groups exhibited significant improvements compared to the CG in terms of writing speed, response time and automation time.

Similarly, Ji et al. (2023) investigated the effect of exergames on attention in 30 children with ADHD. Sixteen participants were randomly assigned to an exergaming group, whilst fourteen were assigned to a cycling exercise group. In the exergaming group, participants played a videogame, Alchemist’s Treasure, on the ExerHeart device, while the cycling group performed exercises on a stationary bike. Both interventions lasted for four weeks, with sessions being held three days a week and lasting 50 min. Participants maintained an intensity of 60–80% of heart rate reserve during the sessions. Outcomes revealed that both exergaming and cycling interventions improved inhibitory control, with improvements in selective and sustained attention, and self-control. However, exergaming resulted in a significantly greater increase in N2 amplitude (a neural marker of attention) compared to cycling.

Additionally, more recent interventions have explored the use of technological platforms to combine physical and cognitive training in an adaptive manner. For example, Zhao et al. (2024) evaluated the efficacy of a digital cognitive–physical intervention called BrainFit for improving EFs in 90 school-aged children with ADHD. The majority of the sample was boys. Participants were randomly assigned to an EG or a CG. The intervention consisted of 12 30 min sessions, which were conducted three times per week on an iPad, and included adaptive modules aimed at developing cognitive and physical skills. Findings showed significant improvements in the EG compared to the CG, with notable reductions in inattention and significant enhancements in EFs, particularly in metacognition and overall EF domains.

## 4. Discussion

### 4.1. Summary of Evidence

Before detailing the specific findings, it is vital to revisit the overarching purpose of this scoping review: to systematically map and reflect upon the existing evidence regarding PA interventions for EFs in children with ADHD. By doing so, this review serves a dual function: it informs our current understanding of which interventions are effective, and it illuminates methodological gaps to guide future, evidence-based educational and clinical practices.

#### 4.1.1. What Types of PA Interventions Have Been Implemented to Enhance EFs in Children with ADHD?

The conclusion that “all types of PA improve the EFs of children with ADHD” (Ziereis & Jansen, 2015) may be overly simplistic. In reality, different types of PA selectively enhance distinct components of EFs, with effectiveness depending on factors such as duration, intensity and modality (Song et al., 2023). Understanding of these nuances is crucial for designing targeted interventions that maximize cognitive benefits in this population.

Short-duration PA interventions stand out due to their ease of implementation in diverse settings, such as classrooms, and the fact that they offer immediate benefits to specific EFs such as response inhibition and verbal working memory (Gawrilow et al., 2016; Miklós et al., 2020; Barudin-Carreiro et al., 2024). However, these effects tend to be transient, especially when compared to those achieved through long-term interventions (Kang et al., 2011; Fedewa et al., 2021; Liang, 2023). Despite requiring a prolonged commitment that may impede adherence in children with ADHD, longer duration interventions tend to generate more robust and sustained improvements across a broader range of EFs, including attention, processing speed, and cognitive flexibility.

In the realm of aerobic exercise, acute sessions are particularly useful for inducing rapid improvements in inhibition and cognitive flexibility, which are beneficial when it comes to preparing children for tasks that demand high cognitive performance (Y. K. Chang et al., 2012; Chuang et al., 2015; Piepmeier et al., 2015; Ludyga et al., 2017; Yu et al., 2020; Tsai et al., 2021). Evidence indicates that moderate-intensity exercise optimizes inhibitory control in acute exercise interventions. On the other hand, prolonged aerobic programs consolidate these improvements and promote positive neurophysiological brain adaptations (Suazo et al., 2019; Verret et al., 2012; Mohammadi-Orangi et al., 2021; Jiang et al., 2022). In this case, sustained adherence is fundamental for maintaining benefits in the long term. Interestingly, while moderate-intensity is preferable in the acute context, high-intensity exercise has also shown promising results in terms of inhibitory control and selective attention during longer lasting interventions (Tsai et al., 2021; Suárez-Manzano et al., 2021, 2022; Sun et al., 2024). Nonetheless, the effectiveness of this type of exercise is not ubiquitous and, instead, varies depending on methodological design and context.

The integration of PA within the school environment offers a valuable opportunity to directly impact academic performance and cognitive skills. PA performed in the classroom setting has been shown to improve response accuracy, reading and arithmetic skills, as well as working memory and sustained attention (Hill et al., 2011; Pontifex et al., 2013; de la Merced-García & Jarauta, 2022). However, not all classroom exercise modalities produce the same effects, which highlights the need for careful designs that consider factors such as novelty and intensity.

Sport and structured physical exercise, which combine playful and social elements, facilitate adherence and are attractive to children with ADHD. Activities like aquatic sports and soccer have consistently shown improvements in inhibitory control, memory and cognitive flexibility (Y. K. Chang et al., 2014; C. J. Huang et al., 2017; Hattabi et al., 2019, 2022; Silva et al., 2020; Jun, 2023; Ludyga et al., 2023; Memarmoghaddam et al., 2016; Z. Huang et al., 2024). Nonetheless, effectiveness is not homogeneous. For example, studies involving judo have produced equivocal outcomes. This suggests that the suitability of a given sport may depend on the specific EFs involved and the individual characteristics of the child.

Beyond traditional PA, relaxation techniques delivered through PA, such as yoga and mindfulness, offer a holistic approach that enhances emotional and cognitive self-regulation, thereby improving sustained attention and memory (Chou & Huang, 2017; Rezaei et al., 2018; Bigelow et al., 2021; Nejatifar et al., 2022). One drawback is that such interventions require specialized instructors; however, their potential to foster self-regulation makes them a valuable tool for children with ADHD.

The innovative approach of combining physical exercise with cognitive training generates synergies that enhance the individual benefits of both modalities. This combination has led to significant and sustained improvements in working memory, cognitive flexibility and inhibitory control (Lee et al., 2017; Wexler et al., 2021; Nejati & Derakhshan, 2021; Nejati, 2021; Liang et al., 2022; Pan et al., 2019; Shams et al., 2021). Despite the complexity involved in designing and implementing these interventions, empirical findings support continued investment in their development.

Finally, exergames represent a technological frontier in PA promotion and cognitive stimulation for children with ADHD. These active video games have produced benefits in terms of attention, visual memory and inhibitory control, in addition to encouraging high adherence due to their playful nature (Garcia-Lara et al., 2016; Weerdmeester et al., 2016; Benzing & Schmidt, 2017, 2019; Benzing et al., 2018; Shema-Shiratzky et al., 2019; S. H. Chang et al., 2022; Ji et al., 2023; Zhao et al., 2024). However, the effectiveness of exergames varies considerably, which emphasizes the importance of high-quality design and rigorous evaluation. The emerging use of adaptive technological platforms opens the door to personalized interventions with the potential to maximize EF benefits.

#### 4.1.2. Which Components of EFs (Inhibitory Control, Working Memory and Cognitive Flexibility) Have Been Most Frequently Targeted or Reported as Improved in Studies on PA Interventions?

Of the core EF domains, inhibitory control, working memory and cognitive flexibility, inhibitory control emerges as the most consistently improved function across studies. Acute aerobic sessions (Y. K. Chang et al., 2012; Yu et al., 2020; Tsai et al., 2021) and structured sports programs (Memarmoghaddam et al., 2016; Suárez-Manzano et al., 2022) reliably led to significant gains in inhibition. This may reflect the particular relevance of motor response inhibition to the demands of physical tasks and cognitive–motor coupling mechanisms activated during exercise.

Working memory improvements were observed, predominantly, following interventions of longer duration and/or those including cognitive training elements (Liang, 2023; Nejati, 2021; Wexler et al., 2021). This suggests that sustained stimulation and task complexity may be necessary to yield transfer to working memory. Cognitive flexibility, although less often a direct target of interventions, improved notably following multi-component interventions (Silva et al., 2020; Benzing & Schmidt, 2019). This indicates that PA engagement may support higher-order EFs when combined with cognitive engagement or novel task demands.

All of the above appears to be aligned with the embodied cognition framework, which suggests that motor activity coupled with cognitive challenges may promote deeper neurocognitive engagement and better transfer (Wilson, 2002).

Recent theoretical advances have expanded this view. For instance, Zou et al. (2025) proposed an integrated model combining embodied cognition and cognitive load theory, highlighting that PA during learning optimizes cognitive resource allocation and enhances working memory and inhibitory control when motor and cognitive demands are balanced. This perspective provides a valuable framework for interpreting how PA interventions that incorporate both movement and cognitive challenge, such as cognitively demanding games or exergames, may yield superior executive outcomes in children with ADHD.

In parallel, Zhang et al. (2025) introduced a dual-process framework explaining how PA can enhance academic performance through both domain-general and domain-specific EFs. This framework suggests that PA fosters not only broad regulatory capacities (e.g., attention and inhibition) but also task-specific processes (e.g., problem-solving or spatial reasoning), which may account for the diverse EF outcomes observed across different intervention types. Integrating this dual-process perspective with current findings could help clarify why certain modalities, particularly those combining aerobic, coordinative, and cognitive elements, appear to produce the most consistent EF improvements.

Interestingly, findings from neurophysiological studies (e.g., Jiang et al., 2022) provide more evidence of behavioral outcomes by showing increased activation in EF-related brain regions, particularly the frontal cortex, following PA engagement. This neurobiological evidence strengthens the argument that PA interventions can modulate the neural substrates responsible for E deficits in ADHD.

#### 4.1.3. What Methodological Features Should Be Considered in Terms of Study Design, Sample Characteristics, Assessment Tools and Outcome Variables?

The reviewed studies exhibit significant methodological heterogeneity, including varied designs (e.g., randomized controlled trials and quasi-experimental approaches), sample sizes with inherently small and often non-representative samples, and PA duration, frequency and type. Although most reported statistically significant improvements in EFs, several limitations prevent firm conclusions and limit the generalizability of findings.

A critical outcome of this scoping review is the identification of significant knowledge gaps. Emphasizing these gaps is essential for advancing the field. A recurring issue to emerge from this research is the wide variability in the age range of study samples and the diversity of cognitive assessment tools, which complicates comparisons between studies and undermines consistency. Additionally, the absence of active CGs or alternative treatments in some studies makes it difficult to distinguish the true effects of PA engagement from natural developmental changes or regression to the mean. These methodological shortcomings are further complicated by differences in medication status, which may introduce confounding effects on cognitive outcomes. Highlighting these methodological shortcomings provides a clear roadmap for future researchers to move beyond exploratory designs toward rigorous, ecologically valid methodologies.

Sample characteristics also pose concerns. Many studies included small sample sizes and were often sex-biased, with a predominant inclusion of boys with ADHD. This limits the external validity of findings and restricts their applicability to broader or more diverse populations. The lack of randomization in some trials, along with the use of passive or inactive CGs, introduces the risk of expectancy effects. Furthermore, the absence of long-term follow-up in most interventions limits our understanding of the durability of the observed cognitive benefits.

Another critical issue is the assessment of EF domains. While some studies focused solely on inhibition (e.g., via the Stroop and Go–No-Go tasks), others examined only two or three EF components, often excluding higher-order skills such as planning and problem-solving. Lack of task variability, limited ecological validity, and possibility of practice effects also reduce the precision of cognitive assessments and hinder the ability to detect real changes over time (Bombín-González et al., 2014).

These limitations highlight the need for more controlled and methodologically rigorous research. The effects of PA on EFs should be interpreted with caution, as many existing studies fail to control for key confounding variables or rely on short-term interventions that lack follow-up assessment. Although PA interventions, ranging from aerobic and structured exercise to sport (e.g., swimming, racket sports and football), mind–body practices like yoga or mindfulness, and even combined PA-cognitive training or exergames, have generally shown efficacy when it comes to improving EFs in primary school children with ADHD, findings must be considered preliminary due to the methodological weaknesses described above.

It is also important to recognize that the observed cognitive effects of PA are typically small (Donnelly et al., 2016). In light of the aforementioned limitations, it remains difficult to establish whether improvements in EFs are directly attributable to PA engagement. While PA has a well-established role in enhancing physical health (e.g., cardiovascular fitness and motor skills) in children with ADHD (López-Sánchez et al., 2014), its impact on cognitive functioning remains a developing area of research. As such, further studies are necessary to clarify the underlying mechanisms and conditions under which PA may benefit EFs in this population.

Future research should adopt longitudinal designs and develop causal models that incorporate mediating (e.g., sleep duration, screen time, cardiovascular training, self-efficacy, motivation, resilience, motor competence, mood, self-esteem and interoception) and moderating variables (e.g., intensity, duration and frequency of PA, task type, and cognitive load). Additionally, models should adjust for potential confounders, such as engagement in cognitively stimulating activities, family socioeconomic status, and personality traits. Researchers should also consider parental influences, including education level, health habits and dietary patterns, which may shape children’s behavior and indirectly affect cognitive outcomes.

Addressing these factors will enable researchers to better isolate the true effects of PA on EFs and move the field towards more robust and conclusive evidence. At present, no definitive conclusions can be drawn regarding the cognitive impact of PA on children with ADHD. Finally, to make sense of this methodological heterogeneity and face future research constructively, this study adopts a reflective practice approach. The methodological reflections woven throughout this review trace back to the foundational concepts of Dewey (1933) and their modern applications across professional contexts (Marshall, 2019). This reflective lens serves as the invisible philosophical strand connecting past empirical evidence with future applications. By explicitly using reflection to evaluate these methodological features, this review not only synthesizes current outcomes but elevates the evaluation process, providing stakeholders with pragmatic, future-facing insights for integrating PA into ADHD management.

### 4.2. Limitations of the Present Scoping Review and Future Perspectives

Despite the strengths of the present scoping review in mapping a wide range of PA-based interventions designed to enhance EFs in children with ADHD, several limitations should be acknowledged. These include the heterogeneity of interventions and assessment tools, exclusion of the gray literature, and potential geographical biases, which may hamper the generalizability and utility of findings. Furthermore, considering the overrepresentation of boys in the included literature (which aligns with epidemiological prevalence but limits external validity), future research should actively recruit more female participants. Exploring potential gender differences in how PA impacts EFs is a critical step toward developing more inclusive, tailored, and effective interventions for all children with ADHD. In addition, future reviews should aim to adopt more structured categorization frameworks, broaden search strategies, incorporate critical appraisal tools, and explore potential moderators (e.g., culture, educational setting and symptom severity) to better inform evidence-based practice. Nevertheless, the present review successfully fulfilled its objective of providing an initial mapping of existing interventions examining the influence of PA on EFs, analyzing and discussing more than 50 studies. Additionally, although no specific section is dedicated to the appraisal of study quality, comprehensive discussion is devoted to methodological considerations. Furthermore, the limitations of each individual study are outlined in detail in Table S1 (Supplementary Material S3).

## 5. Conclusions

The present scoping review provides an updated and comprehensive synthesis of PA interventions designed to enhance EFs in children with ADHD. Findings reveal that both acute and chronic PA interventions yield positive effects on EFs in children with ADHD, particularly on inhibitory control, working memory and cognitive flexibility. Additionally, structured sports (e.g., swimming and football) and multimodal interventions (e.g., combined physical–cognitive training and exergames) showed significant improvements. Intervention efficacy was moderated by exercise intensity, duration, cognitive load, and intervention context (e.g., school-based vs. clinical settings). Among the three core components of executive functioning, improvements in inhibitory control were the most frequently reported across studies, followed by working memory and cognitive flexibility, especially when interventions included both physical and cognitive demands.

Despite these promising outcomes, the review also highlights considerable methodological limitations regarding the existing literature. These include small sample sizes, a lack of standardized cognitive assessments, limited randomization, short intervention durations, and insufficient follow-up. These constraints, along with the heterogeneity of study designs, hinder the ability to draw definitive conclusions about the causal impact of PA on EFs within this population.

Importantly, the present review identifies key gaps in the field and proposes concrete directions for future research, such as the inclusion of long-term follow-ups, use of more ecologically valid assessment tools, and integration of theoretical models and potential mediating and moderating variables. Given the potential of PA as a low-cost, non-pharmacological strategy to support cognitive and behavioral functioning in children with ADHD, more rigorous, theory-driven, and context-sensitive studies are needed to strengthen the evidence base and guide educational and clinical practice. Only through such high-quality research can scientifically grounded practical implications be formulated to inform educational and clinical interventions.

While definitive educational guidelines must await this high-quality research, the current literature offers some preliminary, low-risk strategies that primary school teachers might cautiously explore. For instance, integrating short active breaks between academic tasks could be tentatively used to support attention and inhibitory control. Furthermore, incorporating cognitively engaging physical activities (e.g., coordination challenges) or mind–body practices (e.g., mindfulness) may encourage better self-regulation than purely repetitive tasks. Educators are encouraged to adapt these exploratory strategies flexibly, closely monitoring individual student responses.

## Figures and Tables

**Table 1:** Search terms.

Domain	Descriptors
Physical Activity/Exercise	Physical activity, Exercise, Physical exercise, Aerobic exercise, Isometric exercise, Acute exercise, Exercise training, Physical activity level, Physical fitness, Exergame, Virtual reality exercise, Active video game.
Executive Function/Cognition	Executive function, Executive control, Attention, Cognition, Memory, Problem solving, Cognitive flexibility, Working memory, Inhibitory control, Mental flexibility.
Attention Deficit Hyperactivity Disorder (ADHD)	Attention deficit disorder with hyperactivity, Attention deficit hyperactivity disorder, ADDH, ADHD, Attention disorder, Hyperactivity disorder, Hyperactivity.
Children/Educational Stage	Primary school, Primary education, Elementary school, Junior school, Infant school, Student, Child.
Study Design	Randomized controlled trial, Intervention, Longitudinal study.
Exclusion Criteria	Adults, Systematic review, Review, Meta-analysis.

## Data Availability

No new data were created or analyzed in this study.

## Supplementary Materials

The following supporting information can be downloaded at: https://www.mdpi.com/article/10.3390/bs16050703/s1, Supplementary Material S1: Preferred Reporting Items for Systematic reviews and Meta-Analyses extension for Scoping Reviews (PRISMA-ScR) checklist; Supplementary Material S2: Search equation; Supplementary Material S3: Table S1. Characteristics of studies included.

## Abbreviations

The following abbreviations are used in this manuscript:

ADHD	Attention Deficit/Hyperactivity Disorder
EFs	Executive Functions
PA	Physical Activity
