# Supplementary material for: Effects of Different Physical Activity Approaches on Executive Functions in Primary School Children with ADHD: A Scoping Review with Methodological Reflections
Source: Behav Sci (Basel). 2026 May 4;16(5):703. doi: 10.3390/bs16050703 (PMC13203134; doi:10.3390/bs16050703)
Supplement: Supplementary file 1 [file behavsci-16-00703-s001.zip › Supplementary_material_S2.pdf]

Supplementary material S2

Search Equation

(“physical* activit*” OR “exercis*” OR “physical* exercis*” OR “aerobic* exercis*” OR “isometric* exercis*” OR “acute* exercis*” OR “exercise* train*” OR “physical* activit* level*” OR “physical* fitness” OR “exergam*” OR “virtual realit* exercis*” OR “activ* video* gam*”) AND (“execut* function*” OR “execut* control*” OR “attention” OR “cognition” OR “memory” OR “problem solving” OR “flexibility” OR “working memory” OR “inhibitory control” OR “mental flexibility”) AND (“attention deficit disorder hyperactivity” OR “ADHD” OR “ADDH” OR “attention deficit hyperactivity disorder” OR “attention disorder*” OR “hyperactivity disorder*” OR “hyperactivity”) AND (“primary school*” OR “primary education” OR “elementary school*” OR “junior school*” OR “infant school*” OR “primary school student*” OR “child*”) AND (“randomized control* trial” OR “intervention” OR “longitudinal”) NOT (“adult*”) NOT (“systematic review” OR “review” OR “meta-analysis”)

**Data bases**	**PubMed, Cochrane Library, Web of Science, Scopus, PsycINFO**

PubMed	
Steps	Search terms
#1	(“physical* activit*” OR “exercis*” OR “physical* exercis*” OR “aerobic* exercis*” OR “isometric* exercis*” OR “acute* exercis*” OR “exercise* train*” OR “physical* activit* level*” OR “physical* fitness” OR “exergam*” OR “virtual realit* exercis*” OR “activ* video* gam*”)

#2	(“execut* function*” OR “execut* control*” OR “attention” OR “cogniti*” OR “memory” OR “problem solving” OR “flexibility” OR “working memory” OR “inhibitory control” OR “mental flexibility”)
#3	(“attention deficit disorder hyperactivity” OR “ADHD” OR “ADDH” OR “attention deficit hyperactivity disorder” OR “attention disorder*” OR “hyperactivity disorder*” OR “hyperactivity”)
#4	(“primary school*” OR “primary education” OR “elementary school*” OR “junior school*” OR “infant school*” OR “primary school student*” OR “child*”)
#5	(“adult*”)
#6	(“systematic review” OR “review” OR “meta-analysis”)
Search	(((((#1) AND (#2)) AND (#3)) AND (#4)) NOT (#5)) NOT (#6)
Results	415
Search equation	((("physical* activit*"[All Fields] OR "exercis*"[All Fields] OR "physical* exercis*"[All Fields] OR "aerobic* exercis*"[All Fields] OR "isometric* exercis*"[All Fields] OR "acute* exercis*"[All Fields] OR "exercise* train*"[All Fields] OR "physical* activit* level*"[All Fields] OR "physical* fitness"[All Fields] OR "exergam*"[All Fields] OR "virtual realit* exercis*"[All Fields] OR "activ* video* gam*"[All Fields]) AND ("execut* function*"[All Fields] OR "execut* control*"[All Fields] OR "attention"[All Fields] OR "cogniti*"[All Fields] OR "memory"[All Fields] OR "problem solving"[All Fields] OR "flexibility"[All Fields] OR "working memory"[All Fields] OR "inhibitory control"[All Fields] OR "mental flexibility"[All Fields]) AND ("attention deficit disorder hyperactivity"[All Fields] OR "ADHD"[All Fields] OR "ADDH"[All Fields] OR "attention deficit hyperactivity disorder"[All

	Fields] OR "attention disorder*"[All Fields] OR "hyperactivity disorder*"[All Fields] OR "hyperactivity"[All Fields]) AND ("primary school*"[All Fields] OR "primary education"[All Fields] OR "elementary school*"[All Fields] OR "junior school*"[All Fields] OR "infant school*"[All Fields] OR "primary school student*"[All Fields] OR "child*"[All Fields])) NOT "adult*"[All Fields]) NOT ("systematic review"[All Fields] OR "review"[All Fields] OR "meta-analysis"[All Fields])

Cochrane Library

Steps	Search terms
#1	(“physical* activit*” OR “exercis*” OR “physical* exercis*” OR “aerobic* exercis*” OR “isometric* exercis*” OR “acute* exercis*” OR “exercise* train*” OR “physical* activit* level*” OR “physical* fitness” OR “exergam*” OR “virtual realit* exercis*” OR “activ* video* gam*”)
#2	(“execut* function*” OR “execut* control*” OR “attention” OR “cogniti*” OR “memory” OR “problem solving” OR “flexibility” OR “working memory” OR “inhibitory control” OR “mental flexibility”)
#3	(“attention deficit disorder hyperactivity” OR “ADHD” OR “ADDH” OR “attention deficit hyperactivity disorder” OR “attention disorder*” OR “hyperactivity disorder*” OR “hyperactivity”)
#4	(“primary school*” OR “primary education” OR “elementary school*” OR “junior school*” OR “infant school*” OR “primary school student*” OR “child*”)
#5	(“adult*”)
#6	(“systematic review” OR “review” OR “meta-analysis”)

Results	46
Search equation	(“physical* activit*” OR “exercis*” OR “physical* exercis*” OR “aerobic* exercis*” OR “isometric* exercis*” OR “acute* exercis*” OR “exercise* train*” OR “physical* activit* level*” OR “physical* fitness” OR “exergam*” OR “virtual realit* exercis*” OR “activ* video* gam*”) AND (“execut* function*” OR “execut* control*” OR “attention” OR “cognition” OR “memory” OR “problem solving” OR “flexibility” OR “working memory” OR “inhibitory control” OR “mental flexibility”) AND (“attention deficit disorder hyperactivity” OR “ADHD” OR “ADDH” OR “attention deficit hyperactivity disorder” OR “attention disorder*” OR “hyperactivity disorder*” OR “hyperactivity”) AND (“primary school*” OR “primary education” OR “elementary school*” OR “junior school*” OR “infant school*” OR “primary school student*” OR “child*”) NOT (“adult*”) NOT (“systematic review” OR “review” OR “meta-analysis”)

Web of Science

Steps	Search terms
#1	(“physical* activit*” OR “exercis*” OR “physical* exercis*” OR “aerobic* exercis*” OR “isometric* exercis*” OR “acute* exercis*” OR “exercise* train*” OR “physical* activit* level*” OR “physical* fitness” OR “exergam*” OR “virtual realit* exercis*” OR “activ* video* gam*”)
#2	(“execut* function*” OR “execut* control*” OR “attention” OR “cogniti*” OR “memory” OR “problem solving” OR “flexibility” OR “working memory” OR “inhibitory control” OR “mental flexibility”)

#3	(“attention deficit disorder hyperactivity” OR “ADHD” OR “ADDH” OR “attention deficit hyperactivity disorder” OR “attention disorder*” OR “hyperactivity disorder*” OR “hyperactivity”)
#4	(“primary school*” OR “primary education” OR “elementary school*” OR “junior school*” OR “infant school*” OR “primary school student*” OR “child*”)
#5	(“adult*”)
#6	(“systematic review” OR “review” OR “meta-analysis”)
Results	663
Search Equation	ALL=((“physical* activit*” OR “exercis*” OR “physical* exercis*” OR “aerobic* exercis*” OR “isometric* exercis*” OR “acute* exercis*” OR “exercise* train*” OR “physical* activit* level*” OR “physical* fitness” OR “exergam*” OR “virtual realit* exercis*” OR “activ* video* gam*”) AND (“execut* function*” OR “execut* control*” OR “attention” OR “cognition” OR “memory” OR “problem solving” OR “flexibility” OR “working memory” OR “inhibitory control” OR “mental flexibility”) AND (“attention deficit disorder hyperactivity” OR “ADHD” OR “ADDH” OR “attention deficit hyperactivity disorder” OR “attention disorder*” OR “hyperactivity disorder*” OR “hyperactivity”) AND (“primary school*” OR “primary education” OR “elementary school*” OR “junior school*” OR “infant school*” OR “primary school student*” OR “child*”) NOT (“adult*”) NOT (“systematic review” OR “review” OR “meta-analysis”))

Scopus	
Steps	Search terms
#1	(“physical* activit*” OR “exercis*” OR “physical* exercis*” OR “aerobic* exercis*” OR “isometric* exercis*” OR “acute* exercis*” OR “exercise* train*” OR “physical* activit* level*” OR “physical* fitness” OR “exergam*” OR “virtual realit* exercis*” OR “activ* video* gam*”)
#2	(“execut* function*” OR “execut* control*” OR “attention” OR “cogniti*” OR “memory” OR “problem solving” OR “flexibility” OR “working memory” OR “inhibitory control” OR “mental flexibility”)
#3	(“attention deficit disorder hyperactivity” OR “ADHD” OR “ADDH” OR “attention deficit hyperactivity disorder” OR “attention disorder*” OR “hyperactivity disorder*” OR “hyperactivity”)
#4	(“primary school*” OR “primary education” OR “elementary school*” OR “junior school*” OR “infant school*” OR “primary school student*” OR “child*”)
#5	(“adult*”)
#6	(“systematic review” OR “review” OR “meta-analysis”)
Results	661
Search Equation	(TITLE-ABS-KEY (("physical* activit*" OR "exercis*" OR "physical* exercis*" OR "aerobic* exercis*" OR "isometric* exercis*" OR "acute* exercis*" OR "exercise* train*" OR "physical* activit* level*" OR "physical* fitness" OR "exergam*" OR "virtual realit* exercis*" OR "activ* video* gam*")) AND TITLE-ABS-KEY (("execut* function*" OR "execut* control*" OR "attention" OR "cognition" OR "memory" OR "problem

solving" OR "flexibility" OR "working memory" OR "inhibitory control" OR "mental flexibility")) AND TITLE-ABS-KEY (("attention deficit disorder hyperactivity" OR "ADHD" OR "ADDH" OR "attention deficit hyperactivity disorder" OR "attention disorder*" OR "hyperactivity disorder*" OR "hyperactivity")) AND TITLE-ABS-KEY (("primary school*" OR "primary education" OR "elementary school*" OR "junior school*" OR "infant school*" OR "primary school student*" OR "child*")) AND NOT TITLE-ABS-KEY (("adult*")) AND NOT TITLE-ABS-KEY (("systematic review" OR "review" OR "meta-analysis")))

PsycINFO

Steps	Search terms
#1	(“physical* activit*” OR “exercis*” OR “physical* exercis*” OR “aerobic* exercis*” OR “isometric* exercis*” OR “acute* exercis*” OR “exercise* train*” OR “physical* activit* level*” OR “physical* fitness” OR “exergam*” OR “virtual realit* exercis*” OR “activ* video* gam*”)
#2	(“execut* function*” OR “execut* control*” OR “attention” OR “cogniti*” OR “memory” OR “problem solving” OR “flexibility” OR “working memory” OR “inhibitory control” OR “mental flexibility”)
#3	(“attention deficit disorder hyperactivity” OR “ADHD” OR “ADDH” OR “attention deficit hyperactivity disorder” OR “attention disorder*” OR “hyperactivity disorder*” OR “hyperactivity”)

#4	(“primary school*” OR “primary education” OR “elementary school*” OR “junior school*” OR “infant school*” OR “primary school student*” OR “child*”)
#5	(“adult*”)
#6	(“systematic review” OR “review” OR “meta-analysis”)
Results	436
Search Equation	(“physical* activit*” OR “exercis*” OR “physical* exercis*” OR “aerobic* exercis*” OR “isometric* exercis*” OR “acute* exercis*” OR “exercise* train*” OR “physical* activit* level*” OR “physical* fitness” OR “exergam*” OR “virtual realit* exercis*” OR “activ* video* gam*”) AND (“execut* function*” OR “execut* control*” OR “attention” OR “cognition” OR “memory” OR “problem solving” OR “flexibility” OR “working memory” OR “inhibitory control” OR “mental flexibility”) AND (“attention deficit disorder hyperactivity” OR “ADHD” OR “ADDH” OR “attention deficit hyperactivity disorder” OR ("attention disorders") OR ("hyperactivity disorder") OR “hyperactivity”) AND ((“primary school" OR "primary schooling" OR "primary schools" OR "primary schoolteacher") OR “primary education” OR ("elementary school" OR "elementary schoolchildren" OR "elementary schoolhome" OR "elementary schooling" OR "elementary schoolk" OR "elementary schools" OR "elementary schoolteacher") OR ("junior school" OR "junior schools") OR ("infant school" OR "infant schools") OR “primary school student*” OR “child*”) NOT (“adult*”) NOT (“systematic review” OR “review” OR “meta-analysis”) (Filters: Peer-reviewed articles)
