# Supplementary material for: Effects of Different Physical Activity Approaches on Executive Functions in Primary School Children with ADHD: A Scoping Review with Methodological Reflections
Source: Behav Sci (Basel). 2026 May 4;16(5):703. doi: 10.3390/bs16050703 (PMC13203134; doi:10.3390/bs16050703)
Supplement: Supplementary file 1 [file behavsci-16-00703-s001.zip › Supplementary_material_S3.pdf]

Supplementary Material S3

Table S1. Characteristic of studies included

Table S1. Characteristic of studies included

Reference	Objective (AF and EF)	Sample and Age (range)	Study design	Evaluation method	Main findings (effect of PA on EF)	Limitations	Conclusions
Hill et al. (2011).	- Replicate the finding that a classroom-based exercise regime improves cognitive performance in a more socioeconomically diverse sample.<br><br>- Investigate whether the cognitive benefits of exercise are moderated by BMI or ADHD symptoms.	Total: 522 boys and girls.<br><br>EG: 276.<br><br>CG: 276.<br><br>Scotland.<br><br>8-12 years with a mean age of 9 years 8 months ± 1 year 2 months.	Randomized, double-blind, crossover controlled trial.<br>One group received the exercise intervention in week 1, and the other group in week 2.<br>- EG: 10-15 minutes of moderate-intensity PA led by the teacher (e.g., jogging in place, jumping sequences).<br>- CG: Involved non-physical, enjoyable classroom activities.	- Cognitive Task: Cognitive test battery (CTB) performance.<br><br>- PA Task: Classroom-based exercise (10-15 minutes of moderate-intensity exercises).<br><br>- Physiological Control: BMI classification (normal weight, overweight, or obese) and ADHD symptoms, assessed via parental questionnaire.	- In week 1, no significant difference in CTB performance between the EG and CG (-2.40 [CI -5.99 to 1.19], p = 0.314).<br><br>- In week 2, the exercise group showed a significant improvement in CTB performance compared to the CG (3.85 [CI 0.26-7.44], p = 0.030).<br><br>- Cognitive benefits of exercise were not moderated by sex, ADHD symptoms, or BMI.	- Potential for uncontrolled practice effects and limited generalizability in previous studies.<br><br>- Inability to assess the effect of socioeconomic status due to skewed participation rates.<br><br>- Potential for bias due to missing data, though this was likely minimal.<br><br>- Uncertainty around the true effect size of the exercise intervention, with the confidence interval suggesting a potentially small effect.	- Classroom-based exercise interventions can improve cognitive performance in children.<br><br>- Cognitive benefits emerge specifically in the second testing session, suggesting exercise enhances recall and the ability to utilize learned strategies.<br><br>- Benefits are not influenced by BMI, sex, or ADHD symptoms, indicating exercise could be broadly beneficial and should be incorporated into mainstream education.
Kang et al. (2011).	- Examine attention and cognitive function in children with ADHD through sports therapy.	Total: 28 boys > 80 IQ.<br><br>EG1 (education): 13.<br><br>EG2 (sport): 15.<br><br>South Korea.<br><br>EG1 (education): 8.6 ± 1.2 years.<br><br>EG2 (sport): 8.4 ± 0.9 years.	A 6-week, prospective, randomized controlled trial with two treatment groups.<br><br>- EG1: Received medication and 12 sessions of education around behaviour control.<br><br>- EG2: Received medication and 90 min sports therapy.	- Cognitive task: Attention deficit severity, as measured by the K-ARS-PT scale. Executive function, as measured by the Digit Symbol Test and Trail Making Test Part B.<br><br>- PA task: 90-minute sessions twice a week (Tuesdays and Fridays) for 6 weeks, with activities like aerobic exercise, goal-directed exercises, and rope jumping (EG2).	- Inattention sub-score: Greater improvements in sports therapy group compared to education-only group (p < .01).<br><br>- Digit Symbol Test score: Increase in sports therapy group (p < .01), no change in education-only group.<br><br>- Trail Making Test Part B performance time: Greatly reduced in sports therapy group compared to education-only group (p = .04).	- Small sample size and short study duration make it difficult to generalize outcomes.<br><br>- Repeated cognitive testing over the 6-week period could affect outcomes, although comparing the groups helps mitigate this.<br><br>- The study did not demonstrate the long-term maintenance of effects, and further research with larger samples and longer follow-up is needed.	- Sports therapy improved attention symptoms, especially inattention, in children with ADHD.<br><br>- Sports therapy improved cognitive function and processing speed in children with ADHD.

Table S1. Characteristic of studies included (continued)

Reference	Objective (AF and EF)	Sample and Age (range)	Study design	Evaluation method	Main findings (effect of PA on EF)	Limitations	Conclusions
Chang et al. (2012).	- Examine whether a single bout of aerobic exercise can improve executive function in children with ADHD.	Total: 40 boys and girls (mostly male).<br><br>Taiwan.<br><br>8-15 years, mean 10.43 years.	Randomized controlled trial.<br><br>EG: Exercise group that performed 30 minutes of moderate intensity aerobic exercise.<br><br>CG: A group that watched a video.	-Cognitive task: Stroop test for inhibition, Wisconsin Card Sorting Test (WCST) for set shifting.<br><br>-PA task: 30-minute aerobic exercise session (5 min warm-up, 20 min at 50–70% HRR, 5 min cool-down).	- Acute exercise facilitated performance on the Stroop Colour-Word test, which measures inhibition and cognitive flexibility, in children with ADHD (effect size = 0.57).<br>- Acute exercise improved performance on measures of set-shifting, including non-perseverative errors and categories completed on the Wisconsin Card Sorting Test ($p < 0.05$) in children with ADHD.<br>- Acute exercise particularly benefitted inhibition-related executive function in children with ADHD	- Small sample size and potential confounding factors not accounted for.<br><br>- Potential issues with the CG, such as low motivation or boredom.<br><br>- Limitations of the Stroop Test in measuring inhibition, and the need to focus more on behavioural inhibition.<br><br>- There is a need for more research on the relationship between exercise design, executive function, and the ADHD population.	- Acute moderate-intensity aerobic exercise can improve inhibition and set shifting, two key EF' s that are impaired in children with ADHD.<br><br>- The authors propose that this effect may be due to increased attention, activation of the dorsolateral prefrontal cortex, and exercise-induced dopamine release.<br><br>- While more research is needed, the findings are promising and warrant further investigation into the use of exercise to improve executive function in children with ADHD.
Verret et al. (2012).	- Assess the effects of a moderate-to-vigorous-intensity PA program on fitness, behaviour, and cognitive function in children with ADHD.	Total: 21. (mostly male).<br><br>EG: 10.<br>CG: 11.<br><br>7-12 years (9.1 ± 1.1 years).<br><br>Canada.	RCT.<br><br>- EG: 10-week PA program with 3 sessions per week (45 minutes per session). The program consisted of warm-up, aerobic, resistance, and motor skill exercises, maintaining moderate-to-vigorous intensity, monitored via heart rate.<br><br>- CG: No intervention.	Cognitive Tasks:<br><br>- Tea-Ch (Test of Everyday Attention for Children) for attention and response inhibition.<br><br>Physical Tasks:<br><br>- Fitness (muscular endurance, aerobic capacity).<br><br>- Motor skills (TGMD-2).	Cognition:<br><br>- Attention: The EG group showed significantly better performance on measures of information processing speed and auditory sustained attention compared to the CG ($p = 0.02$ for information processing speed, $p = 0.04$ for auditory attention).	- Difference in recruitment locations and medication use between experimental and CGs.<br><br>- Potential bias from parents and teachers being aware of the treatment.<br><br>- Small sample size and missing data limiting statistical power.	- The 10-week PA program significantly improved muscular capacity, motor skills, behaviour, and cognitive functions, such as attention and information processing, in children with ADHD.<br><br>-These positive effects suggest the clinical relevance of structured PA for children with ADHD, although results should be considered to be preliminary due to methodological limitations.

Table S1. Characteristic of studies included (continued)

Reference	Objective (AF and EF)	Sample and Age (range)	Study design	Evaluation method	Main findings (effect of PA on EF)	Limitations	Conclusions
Pontifex et al. (2013).	- Examine the effect of a single bout of moderate-intensity aerobic exercise on preadolescent children with ADHD.<br><br>- Examine effects using objective measures of attention, brain neurophysiology, and academic performance.	Total: 40 boys and girls.<br><br>United States.<br><br>8-10 years.	Randomized, within-participants, controlled trial. Participants visited the lab on 3 separate days and completed either a 20-minute bout of exercise or 20 minutes of seated reading in a counterbalanced order.<br><br>- EG: 20-minute moderate-intensity aerobic exercise on a treadmill (heart rate maintained between 65-75% of max HR).<br><br>- CG: Seated reading for 20 minutes.	- Inhibitory Control: Modified Eriksen flanker task (measures response accuracy, reaction time, and event-related brain potentials: P3 and ERN).<br><br>- Academic Performance: Wide Range Achievement Test (WRAT3) for reading, spelling, and arithmetic.	- Response Accuracy: Improved in both ADHD and CG after exercise (87.1% after exercise vs. 83.5% after reading, p=0.011, Cohen's d=0.94).<br><br>- Post-Error Slowing: Increased in the ADHD group after exercise (579.4 ms after exercise vs. 500.3 ms after reading, p=0.008, Cohen's d=1.36).<br><br>- Error-Related Negativity (ERN): The difference in ERN amplitude between ADHD and CG was eliminated after exercise (ADHD: -10.8 µV, control: -10.8 µV, p=0.98).<br><br>- Academic Performance: Improved performance on reading comprehension and arithmetic tests in both ADHD and CG after exercise compared to reading.	- The specific components of exercise that optimize its influence on cognition are still unknown.<br>- The study only looked at children with less severe ADHD symptoms, so outcomes may not generalize to those with more severe ADHD or comorbid conditions.<br>- The duration of cognitive benefits from acute exercise is still unclear and may depend on various factors.	- A single bout of moderate-intensity aerobic exercise positively affects neurocognitive function and inhibitory control in children with ADHD.<br><br>- Encouraging PA can be beneficial for neurocognitive function and inhibitory control in children with ADHD.<br><br>- Findings support incorporating short bouts of exercise into the school day as part of a PA program for children with and without ADHD.
Smith et al. (2013).	- Examine the effects of a before-school PA pilot intervention on cognitive, behavioural, motor, and social symptoms in children with ADHD.	Total: 14 boys and girls.<br><br>United States.<br><br>5.2-8.7 years with a mean of 6.7 years.	Pilot, non-controlled, pre-post intervention study conducted over 8 weeks.	- Cognitive tasks: Shape School, Mazes, Finger Windows, Sentence Memory, Numbers Reversed, Response inhibition (Red Light/Green Light, Simon Says).<br><br>- PA task: 30 minutes of moderate-to-vigorous PA before school (4 motor-skill stations, 6 minutes each).	- Significant improvements in response inhibition (Shape School, Red Light/Green Light: medium effects, 0.5–0.8).<br><br>- Teacher-rated behavioural symptoms (e.g., inattention, oppositional behaviour) improved with medium effect sizes.	- Small sample size (n = 14) limits generalizability.<br><br>- No CG or randomization.<br><br>- Focused only on young children with hyperactive/impulsive symptoms and so results may not apply to inattentive type or older children.<br><br>- Further studies with controlled designs are needed.	- A daily before-school PA program may enhance executive functioning, particularly inhibitory control, in young children with ADHD symptoms.<br><br>- Moderate improvements in inhibition tasks and consistent positive ratings from observers support the potential of PA as a promising non-pharmacological intervention for cognitive symptoms of ADHD.

Table S1. Characteristic of studies included (continued)

Reference	Objective (AF and EF)	Sample and Age (range)	Study design	Evaluation method	Main findings (effect of PA on EF)	Limitations	Conclusions
Chang et al. (2014)	- Examine the effects of an 8-week aquatic exercise program, combining aerobic and coordinative activities, on the restraint inhibition component of behavioural inhibition in children with ADHD.	Total: 27 boys and girls with ADHD (mostly male).<br><br>Mean age: 8.78 years.<br><br>EG: 14.<br><br>CG: 13.	Non-randomized controlled trial with a wait-list CG.	- Cognitive task: Go/No Go task to assess response inhibition (accuracy and reaction time for Go and No Go stimuli).<br><br>- PA task: Aquatic program combining aerobic and perceptual-motor exercises.	EG: Significant improvement in inhibitory control. No Go accuracy increased from 88.64% to 94.31% (effect size = 0.9, $p < .05$).<br><br>CG: No change (88.85% to 88.39%, ES = -0.04).	- Small sample size.<br><br>- Non-randomized design.<br><br>- Gender imbalance.<br><br>- No control for PA outside of the program.<br><br>- Lack of broader variability measures relevant to ADHD.	- The aquatic exercise intervention improved inhibitory control, specifically the restraint inhibition component, in children with ADHD.<br><br>- Outcomes suggest that structured, coordinative, and aerobic PA may serve as an effective, non-pharmacological tool to enhance core executive deficits in this population.
Chuang et al. (2015).	- Examine the impact of acute aerobic exercise on Go/No Go task performance (motor inhibition) in children with ADHD.<br><br>- Analyse changes in contingent negative variation (CNV), a marker of attention orienting and response preparation.	Total: 19 children with ADHD (mostly male).<br><br>8-12 years (mean 9.52 ± 1.07 years).<br><br>Taiwan.	Randomized crossover design.<br><br>Each participant completed two conditions in a counterbalanced order:<br><br>1. EG: 30-minute aerobic exercise (treadmill: 5 min warm-up, 20 min at 60% HRR, 5 min cool-down).<br><br>2. CG: 30-minute sedentary control (video-watching).	- Cognitive: Modified Go/No Go task (measuring RT, hit rate, commission and omission errors).<br><br>- PA: Controlled treadmill exercise (monitored with heart rate monitors).<br><br>- Physiological control: EEG (CNV component) recorded from 15 scalp sites (10–20 system), EOG monitored.	Reaction Time (RT): Participants showed significantly shorter RT in the exercise condition compared to control:<br><br>- Exercise: M = 441.60 ms, SD = 62.95<br><br>- Control: M = 470.86 ms, SD = 77.28<br><br>- Hit Rate: No significant difference ($p = .14$).<br><br>- Commission Error Rate: No significant difference ($p = .41$).<br><br>- Omission Error Rate: No significant difference ($p = .26$).<br><br>CNV Amplitude (ERP):<br><br>- CNV 2 (frontal area, No Go stimuli): significantly smaller amplitude after exercise compared to control ($F(1,15) = 5.66$, $p = .031$, $\eta^2 = .27$).<br><br>- In control condition only, frontal CNV 2 was greater for No Go than Go stimuli ($F(1,15) = 10.51$, $p = .005$, $\eta^2 = .41$). This difference was not found in the exercise condition ($F(1,15) = 0.28$, $p = .60$).	- The task may have been too easy—future studies should increase task difficulty.<br><br>- Sample heterogeneity (ADHD subtypes, medication) could confound results.	- Acute aerobic exercise improved response speed and modulated preparatory attention (CNV) in children with ADHD.<br><br>- Exercise eliminated differences in CNV amplitude between Go and No Go stimuli, suggesting a more stable preparatory state.<br><br>- Possible mechanisms include arousal regulation and dopamine modulation

Table S1. Characteristic of studies included (continued)

Reference	Objective (AF and EF)	Sample and Age (range)	Study design	Evaluation method	Main findings (effect of PA on EF)	Limitations	Conclusions
Piepmeier et al. (2015).	- Examine the effect of 20 minutes of moderate intensity exercise on various aspects of executive function performance in children with and without ADHD.	Total: 32 boys and girls (14 diagnosed and 18 no).<br><br>United States.<br><br>8-15 years, mean 10.7 ± 2.27 years.	Randomized controlled within-subjects crossover design. Each participant completed two conditions (exercise and control) in a counterbalanced order.<br><br>EG: 30 minutes (5 min warm-up, 20 min moderate cycling, 5 min cool-down).<br><br>CG: 30 minutes watching a nature documentary.	-Cognitive task: Stroop Task (parts A, B, C): Processing speed and inhibitory control; Tower of London: Planning; Trail Making Test (TMT-A and TMT-B): Set-shifting and speed.<br><br>-Physiological control: HR and RPE monitored during both sessions.<br><br>- PA task: warm-up, moderate cycling and cool-down.	-Performance on the Stroop Task was significantly faster following exercise compared to the control condition, indicating an improvement in inhibitory control and processing speed ($F = 6.11$, $p = .018$, partial $\eta^2 = .17$).<br><br>-No significant effect of acute exercise was observed on planning, as assessed by the Tower of London task ($F = 0.05$, $p = .83$).<br><br>-No significant effects were found on set-shifting, measured by the Trail Making Test ($F = 0.16$, $p = .69$).<br><br>-The effects of acute exercise were not moderated by ADHD diagnosis, suggesting comparable cognitive benefits for both children with and without ADHD.	- Absence of pre-treatment baseline cognitive tests could introduce variability.<br><br>- Heterogeneous use of ADHD medication across participants limits conclusions on medication–exercise interactions.<br><br>- Future studies should compare effects in medicated vs. non-medicated children with consistent medication protocols.	- Acute moderate-intensity aerobic exercise enhanced performance on tasks involving inhibition and processing speed, specifically the Stroop Task, in children regardless of ADHD status.<br>- No benefits were observed for planning or cognitive flexibility, highlighting the selective impact of exercise on specific executive function domains.
Ziereis and Jansen. (2015).	- Determine whether PA has beneficial effects on EF in children with ADHD.<br><br>- Assess whether the type of PA (specific motor training vs. general sports) impacts EF outcomes.<br><br>- Examine both short-term and long-term effects of PA on EF.	Total: 43 boys and girls with ADHD.<br><br>7-12 years with a mean 9.45.	Randomized controlled trial with three groups:<br><br>- EG1 (specific motor skills): ball handling, balance, manual dexterity.<br><br>- EG2 (general sports): general PA without specific focus.<br><br>- CG (wait-list control): no intervention during the study.<br><br>12 weeks, 1 session per week, 60 minutes each (12 sessions total).	- Cognitive tasks: Verbal WM (Digit span forward/backward, letter-number sequencing (HAWIK-IV)), Visuo-spatial WM (Corsi block tapping test).<br><br>- PA task: Movement Assessment Battery for Children (M-ABC 2), including manual dexterity, catching/aiming, static/dynamic balance.	EG1 and EG2 showed significant improvements over CG in verbal working memory:<br><br>- Index-score: $p < .001$, $\eta^2 = .35$.<br><br>- Digit Span Forward: $p < .001$, $\eta^2 = .43$.<br><br>- Letter-Number Sequencing: $p < .05$, $\eta^2 = .15$.<br><br>- Digit Span Backward: $p < .05$, $\eta^2 = .17$.<br><br>- No significant differences between EG1 and EG2, suggesting that both specific and general PA interventions were effective.	- Lack of an additional CG receiving an alternative intervention (e.g. relaxation, stretching).<br>- Only examined 3 executive function domains (verbal/visuospatial working memory, inhibition) and not planning ability.<br>- Used only one task per executive function domain, limiting comparisons with other studies.	- Long-term PA interventions (both specific and general) improved verbal working memory and motor performance in children with ADHD.<br><br>- Type of PA (specific vs. general) did not significantly influence outcomes.<br><br>- PA is a promising tool to support EF development in ADHD populations.

Table S1. Characteristic of studies included (continued)

Reference	Objective (AF and EF)	Sample and Age (range)	Study design	Evaluation method	Main findings (effect of PA on EF)	Limitations	Conclusions
Gawrilow et al. (2016).	- Investigate the effects of a brief PA intervention on executive functioning in children diagnosed with ADHD.	Total: 47 boys and girls (mostly male).<br><br>Germany.<br><br>8.3 to 13.6 years, mean 10.47 years, SD 1.49 years.	Experimental, randomized controlled study.<br><br>Participants were randomly assigned to either a 5-minute PA condition (jumping on a trampoline) or a sedentary control condition (colouring pictures).	- Cognitive task: Attention performance measured by a computerized task. Response inhibition measured by a Go/No-Go task.<br><br>- PA task: jumping on a trampoline for 5 minutes.	Participants who engaged in a 5-minute PA intervention showed significantly better response inhibition ($p < 0.05$) and made significantly fewer errors ($p < 0.05$) on a cognitive task compared to participants in a sedentary control condition.	- Sample size was limited and not designed for additional analyses.<br><br>- The study only included boys with ADHD and did not have a CG without ADHD.	A brief 5-minute bout of PA improved response inhibition and reduced errors on a sustained attention task in children diagnosed with ADHD, compared to a sedentary control condition.
García-Lara et al. (2016).	- Evaluate an intervention program using the Play Attention Interface Box software to train attention in children with ADHD.<br><br>- Apply the intervention in an indigenous community context in Chiapas, Mexico.	Total: 19 boys and girls (mostly males) with ADHD.<br><br>EG: 7.<br><br>CG: 12.<br><br>México.	Experimental design with repeated measures (pre- and post-test).<br><br>- EG: Intervention program combined psychoeducational techniques with brain-activity monitoring and attention training using the Play Attention Interface Box software. Sessions lasted 30–35 minutes, 2–3 times per week for ~4 months.	Cognition task: Attention, impulsivity, and behaviour problems (EEC-R), anxiety (CMAS-R), visual attention, processing speed (SDMT), visual memory and learning (PNMyAV), impulsivity (MFF-20).	EG showed significant improvements in:<br><br>- Attention deficit ($Z = -2.232$, $p < 0.026$)<br><br>- Hyperactivity (LCDSMIV: $Z = -2.388$, $p < 0.017$; EEC-R: $Z = -2.384$, $p < 0.017$)<br><br>- Behavioural problems<br><br>Physiological anxiety ($Z = -2.371$, $p < 0.018$)<br><br>- Social concerns ($Z = -2.201$, $p < 0.028$)<br><br>- Visual attention and memory (SDMT: $Z = -2.197$, $p < 0.028$; PNMyAV: $Z = -2.366$, $p < 0.018$)<br><br>- Cognitive inefficiency (MFF20: $Z = -1.992$, $p < 0.046$)<br><br>CG showed some minor improvements, mainly in visual working memory.	- Small sample size.<br><br>- Need to distinguish ADHD subtypes in future research.<br><br>- Limited scientific evidence on ADHD interventions in Mexico. Results should be generalized with caution.	The intervention program—combining cognitive strategies and the Play Attention Interface Box—was effective at improving attention, working memory, and reducing hyperactive/inattentive behaviours in indigenous children with ADHD.

Table S1. Characteristic of studies included (continued)

Reference	Objective (AF and EF)	Sample and Age (range)	Study design	Evaluation method	Main findings (effect of PA on EF)	Limitations	Conclusions
Memarmoghaddam et al. (2016).	- Examine the effects of a selected exercise program on executive function, specifically cognitive inhibition and behavioural inhibition, in children with ADHD.	Total: 40 boys.<br><br>7-11 years.<br><br>EG: 20.<br>CG: 20.<br><br>Iran.	Randomized controlled trial (not blinded).<br>- EG: Participated in a 24-session exercise program over 8 weeks (3 sessions/week, 90 minutes each) including a warm-up and aerobic exercise, goal-directed exercises, station training, treadmill running, and ball games.<br><br>- CG: Continued regular daily activities without structured exercise intervention.	- Cognitive task: Stroop test (cognitive inhibition), Go-No-Go test (behavioural inhibition).<br><br>- PA task: Structured 90-minute sessions including aerobic exercise, goal-directed games, treadmill running, station circuits, and ball games.<br><br>- Physiological control: Heart rate monitored with Polar watch.	EG:<br>- Cognitive inhibition (Stroop test) significantly improved ($p < .001$), especially in terms of reaction time, correct responses, and reduced errors.<br><br>- Behavioural inhibition (Go-No-Go test) also improved significantly ($p < .001$), with better accuracy and fewer false responses.<br><br>- The intervention explained 86% of variance in Stroop scores and 70% in Go-No-Go scores.<br>Executive function:<br>- Stroop colour-word test improved ($F = 49.64$, $p < .01$, $\eta^2 = 0.32$; sustained at follow-up: $F = 10.59$, $p < .01$, $\eta^2 = 0.40$).<br><br>Improvements were observed in the intervention group compared to the CG during the first phase which were sustained in Group I at 12-week follow-up.	- Only male participants (limits generalizability)<br><br>- Did not analyse differences according to ADHD subtype<br><br>- Excluded children taking ADHD medication<br>- All participants were male, limiting generalizability.<br><br>- Stimulant medication use was not controlled.<br><br>- Participants were from relatively affluent communities.<br><br>- Only parent-reports used for behavioural outcomes.<br><br>- Potential confounding from maturation or classroom experiences.	- A structured, supervised 8-week exercise program improved both cognitive and behavioural inhibition in boys with ADHD.<br><br>- Findings highlight the importance of tailored PA interventions for enhancing EF.<br>- A 12-week table tennis intervention improved motor skills, social behaviours, and EF in children with ADHD.<br><br>- Effects were sustained after 12 weeks.<br><br>- The program is low-cost and viable for community or outpatient implementation.
Pan et al. (2016).	- Assess the effects of a long-term racket-sport intervention (table tennis) on motor skills, social behaviours, and EF in children with ADHD.<br><br>- Examine whether the effects were sustained 12 weeks after the intervention ended (in Group I).	Total: 32 boys with ADHD.<br><br>6-12 years.<br><br>Taiwan.	Randomized controlled crossover trial.<br><br>EG: Intervention during the first 12 weeks.<br><br>12 weeks of table tennis (24 sessions, 70 min/session: 20 min motor skills + 20 min EF games).<br><br>CG: Control during the first 12 weeks, followed by the intervention during the second 12 weeks.	Cognition Task: Stroop Colour and Word Test.	Executive function:<br>- Stroop colour-word test improved ($F = 49.64$, $p < .01$, $\eta^2 = 0.32$; sustained at follow-up: $F = 10.59$, $p < .01$, $\eta^2 = 0.40$).<br><br>Improvements were observed in the intervention group compared to the CG during the first phase which were sustained in Group I at 12-week follow-up.	- All participants were male, limiting generalizability.<br><br>- Stimulant medication use was not controlled.<br><br>- Participants were from relatively affluent communities.<br><br>- Only parent-reports used for behavioural outcomes.<br><br>- Potential confounding from maturation or classroom experiences.	- A 12-week table tennis intervention improved motor skills, social behaviours, and EF in children with ADHD.<br><br>- Effects were sustained after 12 weeks.<br><br>- The program is low-cost and viable for community or outpatient implementation.

Table S1. Characteristic of studies included (continued)

Reference	Objective (AF and EF)	Sample and Age (range)	Study design	Evaluation method	Main findings (effect of PA on EF)	Limitations	Conclusions
Weerdmeester et al. (2016).	- Test the effectiveness and feasibility of using the videogame, Dragon, to decrease ADHD symptoms including inattention, impulsivity, hyperactivity, and motor deficiency.	Total: 73 boys and girls (mostly males) with ADHD.<br><br>EG: 36.<br><br>CG: 37.<br><br>6-13 years (mean age = 9.77 ± 1.74).	Randomized controlled trial (RCT).<br><br>Participants were randomly assigned to either the intervention group (played Dragon) or CG (played Angry Birds).<br><br>6 sessions of gameplay (15 minutes each) over 3 weeks.	Cognition task: Go-No-Go task to assess inhibitory control; observation of impulsivity symptoms.	The intervention group (Dragon) showed a significant decrease in impulsivity; however, both groups (Dragon and Angry Birds) showed deterioration in inhibitory control; increased impulsivity was also observed in the Dragon group during Go-No-Go.	Short intervention duration (only 1.5 hours total).<br>Small sample.<br>Limited to feasibility and short-term effects.<br>Potential task-learning effects.<br>Unexpected negative outcomes in inhibitory control.	While the active video game intervention showed some promise in reducing impulsivity, negative effects on inhibitory control suggest that such interventions require further refinement before being considered effective for enhancing EF in children with ADHD.
Benzing et al. (2017).	- Examine the effects of a cognitively and physically demanding exergame on EF in children with ADHD.	Total: 66 children with ADHD (more boys than girls).<br><br>Age range: 8–12 years.<br><br>Location: Bern, Switzerland.	Randomized controlled trial with parallel-group design and waitlist CG.<br>- EG: 8-week exergame intervention using "Shape UP" on Xbox Kinect, 3 sessions/week, 30 min each.<br>- CG: No intervention during the study period.<br><br>Single-blind: teachers blinded, researchers and parents not blinded.	Cognitive task: Standardized tests assessing inhibition, working memory and cognitive flexibility.<br><br>PA task: Xbox Kinect-based exergame sessions (Shape UP), physically and cognitively demanding tasks.	EG showed significant improvements in EF compared to CG:<br><br>– Inhibition: $F(1,63) = 6.21$, $p = .015$, $\eta^2 = .09$<br>– Working memory: $F(1,63) = 4.17$, $p = .045$, $\eta^2 = .06$<br>– Cognitive flexibility: $F(1,63) = 4.87$, $p = .031$, $\eta^2 = .07$	- Use of a waitlist CG instead of an active comparison group.<br><br>- The exergame was not specifically developed for research purposes, so cognitive engagement may vary across tasks.	- The exergame intervention had significant positive effects on EF and secondary outcomes in children with ADHD.<br><br>- Physically and cognitively demanding exergames may be a promising approach to support EF in this population.

Table S1. Characteristic of studies included (continued)

Reference	Objective (AF and EF)	Sample and Age (range)	Study design	Evaluation method	Main findings (effect of PA on EF)	Limitations	Conclusions
Chou et al. (2017).	- Examine the effects of an 8-week yoga program on sustained attention in children with ADHD, using the Visual Pursuit Test.<br><br>- Examine the effects of an 8-week yoga program on discrimination function in children with ADHD using the Determination Test.	Total: 49 boys and girls (mostly male).<br><br>EG: 24.<br><br>CG: 25.<br><br>Taiwan.<br><br>8-12 years.	Randomized controlled, pre-post study design.<br><br>EG: 8-week yoga program, with 40-minute sessions twice per week. Each session consisted of 10 minutes of warm-up, 20 minutes of yoga activity focused on concentration, balance, attention, breath and body awareness, and a 10-minute cool-down period. The yoga activity was maintained at 50-60% of the participants' maximum heart rate.<br><br>CG: Did not receive any intervention.	- Cognitive tasks: Visual Pursuit Test (Sustained Attention), Determination Test (Discrimination Function).<br><br>- PA task: Flexibility, muscular endurance, power, cardiovascular fitness.<br><br>- Physiological control: Heart rate monitoring during yoga exercise sessions.	- Sustained Attention: The yoga group showed significant improvements in accuracy rate ($p = 0.045$, $d = 0.78$) and reaction time ($p = 0.006$, $d = -1.20$) on the Visual Pursuit Test.<br><br>- Discrimination Function: The yoga group showed a significant improvement in accuracy rate ($p < 0.05$, $d = 1.09$) and faster reaction time ($p = 0.034$, $d = -1.25$) on the Determination Test.<br><br>- The yoga group performed better on both sustained attention and discrimination tests compared to the CG.	- Non-randomized controlled trial design and unbalanced gender proportions (primarily boys).<br><br>- Potential confounding effects of medication use, video games, internet, and television watching.<br><br>- Lack of physical fitness assessment after the 8-week yoga program.<br><br>- Possibility that the observed effects were influenced by the lesser attention paid to the CG.<br><br>- Need for future research on PA outside the intervention and cognitive/behavioural assessments of ADHD symptoms.	- Yoga exercise can be an effective and low-risk alternative treatment for improving cognitive function in children with ADHD.<br><br>- The study is one of few to examine the effects of yoga on cognitive function in ADHD, with promising results as an alternative to stimulant medications.<br><br>- Schools and parents of children with ADHD are encouraged to incorporate yoga into curricula or extracurricular activities.
Huang et al. (2017).	- Examine the effects of an 8-week water aerobics exercise program on resting EEG patterns—specifically theta/alpha ratios—in children with ADHD, as an indirect marker of cognitive and executive functioning.	Total: 32 boys and girls.<br><br>Taiwan.<br><br>5-10 years.<br><br>EG: 16 with a mean age 7.69 ± 1.20 years.<br><br>CG: 16 with a mean age 7.76 ± 1.42 years.	Quasi-experimental, non-randomized, controlled trial.<br><br>EG: An 8-week water aerobics program, with two 90-minute sessions per week, comprising aerobic and perceptual-motor exercises.<br><br>CG: Instructed to avoid regular PA during the study period.	- Cognition task: Resting EEG (theta/alpha ratios and alpha power at frontal and central sites).<br><br>- PA task: 8-week water aerobics program (2x/week, 90 minutes/session, moderate intensity).<br><br>- Control factors: Medication and caffeine restricted 24h prior to EEG; matched groups on ADHD severity, BMI, age.	- Lower theta/alpha ratios in frontal ($p = .005$) and central ($p = .045$) areas for EG.<br><br>- Higher alpha power in frontal ($p = .026$) and central ($p = .033$) regions in EG-<br><br>- These EEG markers suggest improved attentional regulation and executive functioning post-exercise	- Small sample size.<br><br>- Non-randomized design.<br><br>- Gender imbalance.<br><br>- CG may have received less attention.<br><br>- No behavioural or symptom-based outcomes included.	- Aerobic water exercise may lead to neurophysiological improvements in cognitive control, reflected in reduced theta/alpha ratios and increased alpha power.<br><br>- Findings support the potential of PA to modulate EEG-based indicators of EF in children with ADHD, warranting further studies with stronger designs and behavioural outcomes.

Table S1. Characteristic of studies included (continued)

Reference	Objective (AF and EF)	Sample and Age (range)	Study design	Evaluation method	Main findings (effect of PA on EF)	Limitations	Conclusions
Lee et al. (2017).	- Investigate the effects of a 12-week combination exercise program on neuropsychological executive function measures in children with ADHD.<br><br>- Evaluate changes in frontal lobe EEG activity following the exercise intervention.	Total: 18 boys with ADHD.<br><br>EG: 6.<br><br>CG: 6.<br><br>South Korea.<br><br>School students.	Randomized, double-blind, parallel-group controlled trial.<br><br>EG: 12-week combination exercise program (jump rope and ball games), 3 sessions/week, 60 minutes/session (10-min warm-up, 40-min main exercise at 45–75% HRR and 11–16% RPE, 10-min cool-down).<br><br>CG: No intervention.	- Cognition task: Golden's Paediatric Stroop Colour and Word Test (measures Colour-Word and Interference scores).<br><br>- PA task: Combination of aerobic (jump rope) and coordination-based (ball) exercises.<br><br>- Physiological control: EEG (F3 and F4 sites) in task, eyes-open, and eyes-closed conditions; body composition measures.	EEG: Significant increase in beta (β) wave activity in F3 and F4 task conditions ($p<0.05$) in EG compared to CG.<br><br>EF: No significant between-group differences in Stroop Colour-Word or Interference scores.	- Small sample size and pilot study design.<br><br>- No detailed age data or gender diversity.<br><br>- Focus limited to frontal lobe without analysis of broader neural circuits.<br><br>- Limited generalizability due to all-male sample.	- The 12-week combination exercise program led to increased frontal lobe EEG activity in children with ADHD.<br><br>- Improvements in EF were not significantly different between groups.<br><br>- Future studies should explore broader neural networks and larger, more diverse samples.
Ludyga et al. (2017).	- Investigate the acute effects of aerobic and coordinative exercise on inhibitory control and attentional resource allocation in children with and without ADHD.	Total: 36 boys and girls.<br><br>EG: 18 ADHD diagnosed, 13.4 ± 1.5 years.<br><br>CG: 18 without ADHD 13.6 ± 1.6 years.<br><br>Switzerland.	Randomized crossover design with three conditions in counterbalanced order: (1) 20 min aerobic exercise (cycling at 65–70% HRmax), (2) 20 min coordinative exercise (object control, bilateral coordination), (3) 20 min control (watching a documentary).	-Cognition task: Reaction time (RT) and accuracy on Flanker task, P300 amplitude and latency (attention allocation).<br><br>-Physiological monitoring: HR and RPE during exercise.<br><br>- PA task: cycling or coordinative exercise.	- EG: Both aerobic and coordinative exercise led to significantly increased P300 amplitude ($p < .05$) and reduced reaction time ($p < .05$) compared to the CG, indicating improved inhibitory control and attentional allocation.<br>- EG: Aerobic exercise produced larger increases in P300 amplitude ($F(2, 68) = 4.32$, $p = .017$, $\eta^2 = .11$) and greater reductions in reaction time ($F(2, 68) = 3.89$, $p = .024$, $\eta^2 = .10$) than coordinative exercise.<br><br>- CG: No significant differences were observed between exercise modalities in either P300 amplitude or reaction time ($p > .05$).<br><br>- Accuracy on the Flanker task did not significantly differ between conditions or groups ($p > .05$).	- Possible cross-over effects from the crossover design.<br><br>- EEG signal quality could be affected by post-exercise sweating artefacts.<br><br>- Baseline group differences in P300 amplitude may confound interpretation.<br><br>- All ADHD participants were medicated with methylphenidate, limiting generalizability to unmedicated children or other subtypes.	- A single session of aerobic or coordinative exercise improved inhibitory control and attentional processing in both children with and without ADHD.<br>- However, aerobic exercise was especially effective in children with ADHD, producing larger improvements in both neural (P300) and behavioural (reaction time) measures.<br>- Findings suggest that acute aerobic exercise can be a beneficial preparatory strategy for cognitive tasks requiring attention and inhibition, particularly for children with ADHD.

Table S1. Characteristic of studies included (continued)

Reference	Objective (AF and EF)	Sample and Age (range)	Study design	Evaluation method	Main findings (effect of PA on EF)	Limitations	Conclusions
Benzing et al. (2018).	- Investigate the effects of an acute bout of PA (exergaming) on multiple aspects of EF (inhibition, switching, and visual working memory) in children with ADHD.	Total: 46 diagnosed with ADHD (mostly male).<br><br>EG: 23.<br>CG: 23.<br><br>8-12 years, mean age 10.48 ± 1.38 years.<br><br>Switzerland.	Randomized, double-blind, controlled, parallel-group design.<br><br>- EG (exergaming group): played a physically and cognitively engaging exergame (" Shape Up" on Xbox Kinect) for 15 minutes.<br><br>- CG: watched a documentary for the same duration.	Cognitive tasks: - Flanker task (inhibition and switching), Colour Span Backwards task (visual working memory).<br><br>Physiological control: - Heart rate (Polar Team 2 Pro system), OMNI scale of perceived exertion.	Exergaming group showed shorter reaction times in:<br><br>- Incongruent Flanker trials ($p = .022$, $\eta^2_p = 0.117$)<br><br>- Switching Flanker trials ($p = .024$, $\eta^2_p = 0.113$)<br><br>- Global switch costs ($p = .041$, $\eta^2_p = 0.094$)<br><br>No significant group differences in accuracy or visual working memory performance.	- Small sample size impeded subgroup analyses.<br><br>- High proportion of males limited generalizability.<br><br>- Effects in the absence of medication are unclear.<br><br>- Exercise intensity was estimated and not directly measured.<br><br>- Inability to separate physical vs. cognitive effects of exergaming.	A single 15-minute session of moderate-to-vigorous exergaming improved reaction times in inhibition and switching (but not accuracy or working memory) in children with ADHD. This suggests acute PA may selectively enhance specific EF and could be a useful tool in this population.
Rezaei et al. (2018).	- Evaluate the effects of a neurofeedback (NFB) intervention on sustained attention in children with ADHD.<br><br>- Evaluate the effects of a yoga intervention on sustained attention in children with ADHD.<br><br>- Compare the effects of NFB and yoga interventions on memory and cognitive activity in children with ADHD.	Total: 21 boys and girls with ADHD.<br><br>EG1 (neurofeedback): 7.<br><br>EG2 (yoga): 7.<br><br>CG: 7.<br><br>Iran.	Randomized, controlled, parallel-group design. Participants were randomly assigned to one of three groups: neurofeedback, yoga, or control.<br><br>- EG1 (neurofeedback): Theta/beta training at the Cz site using a dual-channel device.<br><br>- EG2 (yoga): Included meditation, relaxation, deep breathing, physical and imagination exercises, designed for 4–12-year-old children.<br><br>- CG: No intervention.	- Cognition tasks: Continuous Performance Test (CPT): correct responses, reaction time, commission errors, omission errors, WISC-R subtests: arithmetic, letter-number sequencing, coding.<br><br>- PA task: Yoga intervention sessions including physical postures, breathing exercises, and guided imagery.<br><br>- Physiological control: Baseline EEG assessment in neurofeedback group.	Neurofeedback vs Control:<br>- Improved omission errors ($p = 0.006$, $F = 17.147$), correct responses ($p = 0.007$, $F = 16.131$), and digit memory ($p < 0.001$, $F = 48.344$).<br>- No significant changes in response time, response errors, math, or coding.<br><br>Yoga vs Control:<br>- Improved response errors ($p = 0.001$, $F = 43.321$), omission errors ($p = 0.008$, $F = 15.523$), correct responses ($p < 0.001$, $F = 47.693$), digit memory ($p = 0.004$, $F = 0.059$), and coding ($p = 0.015$, $F = 11.238$).<br>- No significant change in response time or math.<br><br>Neurofeedback was more effective than yoga in reducing response errors and improving correct responses.	- Lack of control over yoga exercise intensity and execution.<br><br>- Lack of control over medication use by participants.	- Neurofeedback and yoga interventions significantly improved sustained attention and memory in children with ADHD compared to the CG.<br><br>- Both interventions should be considered as complementary, not stand-alone, treatments.<br><br>- The benefits of yoga may relate to neurochemical effects like reduced cortisol and increased dopamine/serotonin.

Table S1. Characteristic of studies included (continued)

Reference	Objective (AF and EF)	Sample and Age (range)	Study design	Evaluation method	Main findings (effect of PA on EF)	Limitations	Conclusions
Benzing et al. (2019).	Investigate the effects of a cognitively and physically demanding exergaming intervention on EF.	Total: 51 children with ADHD (mostly male).<br><br>8-12 years with a mean age of 10.43 ± 1.37 years.<br><br>Switzerland.	Parallel group randomized controlled trial (RCT).<br><br>EG: The exergaming intervention involved 8 weeks of training, 3 times a week, 30 minutes per session using the Xbox Kinect "Shape UP" game, combining physical and cognitive demands.<br><br>CG: No intervention.	Cognitive Tasks<br><br>- Inhibition: Modified Simon task (mean reaction time).<br><br>- Switching: Modified Flanker task (mean reaction time on switching trials).<br><br>- Updating: Modified colour span backward task (sum of correct responses).<br><br>Physiological Control<br><br>- Heart rate, perceived physical exertion, perceived cognitive engagement.<br><br>- Training duration, valence during training, enjoyment of the activity.	EF<br><br>- Inhibition: Faster reaction times in the exergaming group compared to control ($p = 0.049$, Cohen's $d = 0.58$).<br><br>- Switching: Faster reaction times in the exergaming group compared to control ($p = 0.029$, Cohen's $d = 0.65$).<br><br>- Updating: No significant effects on updating executive function.	- High dropout rate in the exergaming group due to lack of incentives or feedback.<br><br>- Use of a waitlist CG instead of an active CG, increasing the risk of the Hawthorne effect.<br><br>- Non-customized exergame that may not have been optimized for children with ADHD.<br><br>- Small sample size, limiting the ability to investigate relationships between variables.<br><br>- Reliance on self-reported data from children' s diaries instead of objective performance data.<br><br>- No assessment of comorbidities or detailed monitoring of medication changes.	- Exergaming improved EF (inhibition and switching) and motor abilities in children with ADHD, which may positively affect their psychopathology.<br><br>- Exergaming could serve as a home-based intervention for children with ADHD, but customized exergames are needed to maximize benefits.
Pan et al. (2019).	- Examine effects of the intervention on EF in children with ADHD.	Total: 60 boys.<br><br>- EG1 (training group): 15 with ADHD.<br><br>- EG2 (non-training group): 15 with ADHD.<br><br>- CG (typically developing non training group): 30 without ADHD.<br><br>Taiwan.<br><br>7-12 years.	Randomized, controlled, parallel-group pilot study.<br><br>- EG1: The intervention lasted 12 weeks, with 24 sessions (2 per week, 70 minutes each). Sessions included 20 minutes of basic table tennis training, 20 minutes of EF training through table tennis tasks, and 20 minutes of group games and conditioning exercises.<br><br>- EG2 and CG: No intervention.	- Cognition tasks: Stroop Colour and Word Test (inhibitory control), Wisconsin Card Sorting Test (WCST) (cognitive flexibility and EF components).	- Stroop Test (inhibitory control): Significant improvement in the Colour-Word condition in EG1 compared to CG and EG2 ($F(2, 57) = 6.84$, $p < .01$, $\eta^2 = .20$).<br><br>WCST: Total correct responses: significantly improved in EG1 ($F(2, 57) = 4.58$, $p < .05$, $\eta^2 = .14$). Perseverative errors and categories completed: no significant differences between groups ($p > .05$).	- Limited scope of EF and motor assessments.<br><br>- No control over medication use.<br><br>- Small and homogeneous (all male) sample.<br><br>- ADHD subtypes not analysed separately.<br><br>- Lack of active CG limits causal interpretations.	- The 12-week table tennis intervention improved motor skills and EF (especially inhibitory control and cognitive flexibility) in children with ADHD.<br><br>- However, due to design limitations, further research is needed before generalizing findings.

Table S1. Characteristic of studies included (continued)

Reference	Objective (AF and EF)	Sample and Age (range)	Study design	Evaluation method	Main findings (effect of PA on EF)	Limitations	Conclusions
Shema-Shiratzky et al. (2019).	- Examine the feasibility and efficacy of combined motor-cognitive training using virtual reality to enhance behaviour, cognitive function, and dual-tasking in children with ADHD.	Total: 14 non-medicated children with ADHD (mostly males).<br><br>8–12 years (mean = 9.4 ± 1.3).<br><br>Israel.	Single-group pre-test/post-test design.<br><br>VR-based treadmill training for 6 weeks (18 sessions, 3×/week, 30–60 min).<br><br>Participants walked on a treadmill while navigating virtual obstacles (motor-cognitive dual-task).	- Cognitive task: NeuroTrax computerized battery (attention, memory, executive function).<br>- PA task: Walking on a treadmill while navigating VR obstacles (dual-task setting).<br>- Physiological control: Gait analysis via instrumented walkway and accelerometer.	- Memory Index Score: Improved significantly post-training ($p = .001$, $d = 0.86$) and at 6-week follow-up ($p = .003$, $d = 1.40$).<br>- Restless-impulsive behaviour: Trend ($p = .055$, $d = 0.62$)	- No CG.<br>- Small sample size and high dropout.<br>- Participants had relatively mild ADHD symptoms (possible ceiling effects).	- VR training was feasible and improved memory, behavioural symptoms, and gait regularity in children with ADHD.<br>- Promising as a non-pharmacological adjunct therapy.<br>- Further RCTs needed to confirm efficacy compared to pharmacological and other interventions.
Suazo et al. (2019).	- Evaluate whether PA improves the cognitive function of school-aged patients with ADHD.<br><br>- Evaluate the effect of a 6-week medium-to-high intensity (70% VO_2max) PA program (2 sessions of 1 hour per week) on the quality of attention and impulsivity in 24 participants diagnosed with ADHD.	Total: 24 boys and girls (mostly male) with ADHD.<br><br>EG: 10.31 ± 3.15 years.<br><br>CG: 10.45 ± 2.58 years.<br><br>Spain.	Randomized controlled trial.<br><br>EG: 6-week intervention: 2 sessions/week, 1 hour/session.<br><br>CG: CG continued regular school activities.	- Cognitive task: Magallanes Visual Attention Scale (EMAV 1 and 2) to assess: Quality of attention, sustained attention, impulsivity (measured via omissions and errors).<br><br>- PA task: Aerobic PA program: 6 weeks, 2 sessions/week, 1 hour/session, Moderate-to-high intensity (60% of VO_2max), Warm-up (10 min), aerobic games (30 min), cool-down (5 min), supervised by a PA specialist, Borg RPE scale used to estimate intensity.	- Quality of attention: significant improvement in the EG ($p = .005$, moderate-large effect size).<br><br>- Sustained attention: significant improvement in the EG ($p = .021$, moderate effect size).<br><br>- No significant effects on impulsivity.	- Intensity not directly measured (no VO_2max, HR, or cortisol monitoring).<br><br>- Cannot confirm that cognitive improvements were due to physiological changes from exercise.<br><br>- Suggests further research monitoring heart rate and salivary cortisol to demonstrate the physiological modifications from the aerobic activities.	- PA improves attention in children with ADHD.<br><br>- A directed PA intervention in schools could be a complementary treatment to medication for patients with attention problems.<br><br>- Increasing the hours of PA in schools and having sports professionals direct these activities may improve the clinical evolution of patients with ADHD.

Table S1. Characteristic of studies included (continued)

Reference	Objective (AF and EF)	Sample and Age (range)	Study design	Evaluation method	Main findings (effect of PA on EF)	Limitations	Conclusions
Miklós et al. (2020).	- Examine the effects of a single bout of moderately intense exercise on attention and EF in treatment-naïve ADHD children, medicated ADHD children, and typically developing children.	Total: 150 boys and girls (mostly male).<br><br>EG1: 50 treatment-naïve ADHD.<br><br>EG2: 50 medicated ADHD.<br><br>CG: 50 typically developing.<br><br>Hungary.<br><br>6-12 years.	A randomized, controlled trial with a parallel group design.<br><br>Random assignment to 20-minute moderate intensity PA while watching a cartoon video or 20-minute control condition of seated while watching a cartoon video.	- Cognitive task: Attention and executive function performance as measured by the 5 KiTAP subtests: alertness, distractibility, divided attention, flexibility, and reaction control (inhibition, go/no-go)<br><br>- PA: 20-minute moderate intensity PA (60-80% of maximum heart rate) while watching a cartoon video.	- EG1 and EG2: PA intervention significantly improved performance on 2 out of 15 measures compared to CG.<br><br>- EG1 showed smaller improvements over time compared to EG2 and CG, suggesting that the PA intervention was less effective for this group.<br><br>- EG1: PA intervention led to significantly greater improvements compared to the CG on one measure (number of total errors and errors with distractor in the distractibility task).	- Significant age differences between the different groups.<br><br>- The intervention was delivered at different times of the day for the CG vs. EGs.<br><br>- Exclusion of certain psychiatric conditions from the CG but not the EGs.<br><br>- Significant diagnostic differences between the medicated and non-medicated ADHD groups.	The study found some positive effects of 20 minutes of moderate-intensity PA on attention and executive function in children with ADHD, regardless of whether they were taking medication or were treatment-naïve.
Silva et al. (2020).	- Investigate the effects of a swimming program on mental health parameters (depression, stress, anxiety), cognitive function (cognitive flexibility, selective attention), and motor coordination (balance, coordination) in children with ADHD.	Total: 20 participants, both genders.<br><br>GE: 10 with a mean age of 12.2 ± 2.0 years.<br><br>CG: 10 with a mean age of 12.0 ± 1.0 years.<br><br>Brazil.	RCT with a pre-post design.<br><br>EG: 8-week swimming program with 2 sessions per week.<br><br>CG: No intervention.<br><br>The swimming program consisted of 45-minute sessions, including 5 minutes of warm-up/stretching, 25 minutes of swimming exercises, 10 minutes of recreational swimming, and 5 minutes of cooldown/stretching.	Cognitive Tasks:<br><br>- Trail Making Test (cognitive flexibility)<br><br>- Cancellation Attention Test (selective attention)<br><br>Physical Tasks:<br><br>- Balance test, lower limb coordination, speed/agility, spatial-temporal coordination	Cognition:<br><br>- Significant improvement in cognitive flexibility (109 ± 10 scores, p=0.042)<br><br>- Significant improvement in selective attention (104 ± 9 scores, p=0.047)	- No aerobic conditioning test to measure improvements in aerobic fitness<br><br>- Lack of measurement of the intensity of swimming sessions	The 8-week swimming program significantly improved mental health parameters (depression and stress), cognitive function (cognitive flexibility and selective attention), and motor coordination (lower limb coordination, laterality, flexibility, abdominal resistance) in children with ADHD.

TableS1. Characteristic of studies included (continued)

Reference	Objective (AF and EF)	Sample and Age (range)	Study design	Evaluation method	Main findings (effect of PA on EF)	Limitations	Conclusions
Yu et al. (2020).	- Evaluate the immediate effects of acute moderate-intensity aerobic exercise on inhibitory control, neuroelectric activity (N2 and P3), and heart rate variability (HRV) in children with ADHD.	Total: 30 children diagnosed with ADHD (mostly male).<br><br>EG: 15.<br>CG: 15.<br><br>8-12 years.<br><br>Taiwan.	A randomized, double-blind, placebo-controlled trial design.<br><br>EG: Acute moderate-intensity aerobic exercise protocol involving a 5-minute warm-up, 20 minutes of treadmill exercise at 60-70% heart rate reserve, and a 5-minute cool-down.<br><br>CG: 30 minutes of quiet video watching.	Cognitive Tasks:<br><br>- Modified flanker task to assess inhibitory control (response accuracy and reaction time).<br><br>- Event-related brain potentials (ERPs): N2 and P3 components during the flanker task.<br><br>Physical Tasks:<br><br>- Moderate-intensity aerobic exercise protocol (treadmill) and a video-watching session as the control condition.<br><br>Physiological Control:<br><br>- Heart rate variability (HRV), including low-frequency (LF-HRV), high-frequency (HF-HRV), and LF/HF ratio.	Acute aerobic exercise led to significant improvements in inhibitory control, including:<br><br>- Increased response accuracy on the flanker task (90.4% vs 83.7%, p=0.001).<br><br>- Larger N2 amplitude (-11.4 µV vs -9.5 µV, p=0.012), indicating improved conflict detection.<br><br>- Shorter N2 latency (286.9 ms vs 294.6 ms, p=0.029), suggesting improved processing speed for conflict resolution.	- The sample was predominantly male, limiting generalizability to girls.<br><br>- The study sample had a high socioeconomic status (SES), which may not reflect the broader ADHD population.<br><br>- The study had an unbalanced distribution of ADHD subtypes, and the majority had the combined type, which limits understanding of subtype-specific effects.<br><br>- Some participants were taking ADHD medications, but the small sample size limited ability to analyse the impact of medication use.	- Acute moderate-intensity aerobic exercise improved inhibitory control and conflict detection in children with ADHD for up to 60 minutes post-exercise.<br><br>- However, it did not have lasting effects on heart rate variability or autonomic nervous system function. This suggests that while aerobic exercise may benefit cognitive performance in ADHD, it may not influence physiological regulation in the long term.
Bigelow et al. (2021).	- Investigate the impact of acute exercise and mindfulness meditation on executive functioning in children and youth with ADHD.<br><br>- Investigate the impact of acute exercise and mindfulness meditation on psycho-emotional well-being in children and youth with ADHD.<br><br>- Directly compare the efficacy of acute exercise and mindfulness meditation in supporting executive functioning and psycho-emotional well-being in children and youth with ADHD.	Total: 16 boys and girls with ADHD (mostly males).<br><br>Canada.<br><br>10-14 years (11.38 ± 1.5).	Pre-post-test, counterbalanced within-subjects design. Experimental and control sessions separated by 1 week.<br><br>Each participant completed three conditions in randomized order: (1) 10 minutes of moderate-intensity cycling (65–85% max HR), (2) 10 minutes of guided mindfulness meditation (via smartphone app), (3) 10 minutes of reading control (age-appropriate material).<br><br>Familiarization session included.	- Cognition task:<br>Inhibitory control: Flanker task, Working memory: 2-back task, Task-switching: Dimensional Change Card Sort task (DCCS).<br><br>- PA task: 10 minutes of moderate-intensity cycling (exercise condition).<br><br>- Physiological control: Heart rate, BMI, perceived exertion recorded.	Mindfulness meditation:<br><br>- Significantly improved inhibitory control ($p = 0.01$, $d = 0.86$ immediate; $p = 0.04$, $d = 0.57$ delayed),<br><br>- Improved working memory ($p = 0.01$, $d = 0.55$ immediate; $p < 0.001$, $d = 0.78$ delayed),<br><br>- Improved task-switching ($p = 0.04$, $d = 0.56$ immediate).<br><br>Acute exercise:<br><br>- No improvements in executive functioning,<br><br>- But improved positive mood and general self-efficacy (stats not reported).	- No neurotypical CG.<br><br>- ADHD diagnosis methods varied.<br><br>- No trait mindfulness assessment or manipulation check.<br><br>- Did not analyse the influence of ADHD medication use.	- Acute mindfulness meditation improved executive functioning (inhibitory control, working memory, task-switching) in children and youth with ADHD.<br><br>- Acute exercise did not affect executive functioning but improved positive mood and general self-efficacy.<br><br>- These interventions may be personalized based on individual needs in ADHD treatment planning.

Table S1. Characteristic of studies included (continued)

Reference	Objective (AF and EF)	Sample and Age	Study design	Evaluation method	Main findings (effect of PA on EF)	Limitations	Conclusions
Chan & Ho (2021).	- Analyse the effects of an 8-week moderate PA program on the reaction ability of children with ADHD.<br><br>- Examine the impact of an adapted PA program, combining aerobic and perceptual-motor exercises, on reaction performance.	Total: 37 boys and girls with ADHD (mostly male).<br><br>EG: 21.<br><br>CG: 16.<br><br>8-10 years.<br><br>Taiwan.	Controlled pre-post intervention pilot study.<br><br>EG: An 8-week adapted PA program (2 sessions/week, 60 minutes each). Each session included a 5-min warm-up, 20 min of moderate-intensity interval training, 20 min of perceptual-motor tasks, 10 min group games, and 5-min cool-down.<br><br>CG: No intervention during the study period.	Cognitive task:<br><br>- Simple Reaction Time (SRT).<br><br>- Choice Reaction Time (CRT).<br><br>- Variability of SRT and CRT.<br><br>PA task:<br>- Adapted PA program combining aerobic and perceptual-motor exercises.	Significant reduction in CRT in the EG (808 ± 243 ms to 714 ± 197 ms, $p < 0.05$); no change in the CG.<br><br>Boys: CRT reduced from 764 ± 277 ms to 685 ± 228 ms ($p < 0.05$).<br><br>Girls: CRT reduced from 918 ± 56 ms to 788 ± 51 ms ($p < 0.05$).<br><br>Significant increase in SRT variability in CG boys (157 ± 56 to 215 ± 95, $p < 0.05$); no significant change in the EG.<br><br>No significant changes in CRT variability for either group.	- Pilot nature of the study and small sample size.<br><br>- No multidisciplinary comparison (e.g., medication or behavioural therapy).<br><br>- No long-term follow-up or generalization to real-world tasks.<br><br>- Lack of direct executive function measures beyond reaction time.	- The adapted PA program improved reaction performance and visual attention in children with ADHD.<br><br>- PA, particularly moderate-intensity, may enhance information processing and reduce variability in reaction tasks.<br><br>- Future studies should explore how exercise design affects broader EF and social skills in this population.<br><br>- Authors advocate incorporating such programs into adapted Physical Education curricula for children with ADHD.
Fedewa et al. (2021).	- Examine the effectiveness of a daily PA intervention compared to a sedentary control condition for improving attention, behaviour, and executive function in children with ADHD.<br><br>- Evaluate the potential moderating effects of the intensity and duration of PA on children's attention, behaviour, and executive function.	Total: 59 boys and girls with ADHD.<br><br>EG: 35.<br><br>CG: 24.<br><br>5-8 years.<br><br>United States.	RCT.<br><br>EG: structured moderate-to-vigorous PA, 30 minutes/session, 3 days/week, for 16 weeks. Activities conducted by trained researchers, both indoor and outdoor (though mostly indoors due to constraints).<br><br>CG: sedentary game-based play, same schedule.	Cognitive tasks:<br><br>- ADHD Rating Scale-5 (parent and teacher reports): measures inattention and hyperactivity/impulsivity<br><br>- Behaviour Rating Inventory of Executive Functioning-2 (BRIEF-2) (parent and teacher reports): assesses EF domains (e.g., working memory, inhibition)<br><br>PA tasks:<br><br>- Physical activity intensity and duration monitored using Adidas Zone Accelerometer and Heart Rate Monitor<br><br>Physiological control:<br><br>- Heart rate measured as percentage of heart rate max<br><br>- BMI calculated from height and weight	- No significant effects of the intervention on executive functioning or ADHD symptoms ($p > .05$), based on either parent or teacher reports.<br><br>Moderating effects of activity intensity (teacher report):<br><br>- More time in moderate PA was associated with worse EF ($\beta = -0.40$, $p = .007$).<br><br>- More time in vigorous PA was associated with better EF ($\beta = 0.35$, $p = .031$).<br><br>- More time in light PA was also associated with better EF ($\beta = 0.41$, $p = .010$).	- Small sample size, especially in the at-risk ADHD subgroup.<br><br>- Lack of direct behavioural assessments (reliance on parent and teacher rating scales).<br><br>- Intervention was often conducted indoors instead of outdoors as planned.<br><br>- Potential mismatch between the types of PA activities and targeted EF domains.<br><br>- Possible baseline differences between groups despite randomization.	- The PA intervention did not produce significant improvements in ADHD symptoms or executive functioning.<br><br>- Some associations between time spent in light or vigorous PA and improved teacher-reported EF suggest potential benefits of activity intensity variation.<br><br>- More research is needed using direct behavioural EF tasks, larger ADHD-risk samples, and optimized activity designs.<br><br>- PA could still be a promising non-pharmacological tool for improving classroom behaviour and self-regulation, but further evidence is required.

Table S1. Characteristic of studies included (continued)

Reference	Objective (AF and EF)	Sample and Age (range)	Study design	Evaluation method	Main findings (effect of PA on EF)	Limitations	Conclusions
Mohammad (2021).	- Investigate the effectiveness of executive function training based on motor activity on working memory and sustained attention of children with ADHD.	Total: 20 children with ADHD.<br><br>EG: 10.<br>CG: 10.<br><br>9-12 years.<br><br>Kermanshah (Iran).	Quasi-experimental pre-post design with CG.<br><br>The EG received a 12-week intervention (2 sessions/week, 70 minutes/session) involving motor activity-based executive function training conducted in a gymnasium.	Cognitive tasks:<br><br>- N-back task for working memory<br><br>- Continuous Performance Test (CPT) for sustained attention	- Significant improvement in working memory in the EG compared to the CG ($p \leq 0.001$), with 92% of variance attributed to the intervention.<br><br>- Significant improvement in sustained attention in the EG compared to the CG ($p \leq 0.001$), with 89% of variance attributed to the intervention.	Not explicitly reported by the authors.	Executive function training based on motor activity significantly improved working memory and sustained attention in children with ADHD. Findings support its use as an effective intervention for improving cognitive functions in this population.
Mohammadi-Orangi et al. (2021)	- Investigate the effects of aerobic exercise with music on anxiety, depression, motor proficiency, and cognitive ability (working memory, perceptual reasoning, and processing speed) in boys with ADHD.<br><br>- Analyse changes in these outcomes after an 8-week aerobic exercise program using nonlinear pedagogy.	Total: 36 boys with ADHD.<br><br>EG: 18 with a mean age of 11.32 ± 1.00 years.<br><br>CG: 18 with a mean age of 11.26 ± 0.92 years.<br><br>Tehran, Iran.	Quasi-experimental, randomized, pre-test-post-test CG design.<br><br>EG: 8-week intervention, 3 sessions per week, 60 minutes per session.<br><br>CG: Continued routine school activities with linear training methods.	- Cognitive tasks: WISC-IV subtests for working memory, perceptual reasoning, processing speed.<br><br>- PA task: Aerobic training program with music, using nonlinear pedagogy. 3 sessions/week, 60 min/session for 8 weeks.	Significant improvements in the EG compared to the CG:<br><br>- Working memory: +6.50 units ($p < .001$).<br><br>- Perceptual reasoning: +6.23 units ($p < .001$).<br><br>- Processing speed: +8.34 units ($p < .001$).	- No female participants included.<br><br>- No comparison with other types of interventions (e.g., aerobic exercise without music).<br><br>- The CG's activity was not fully monitored with possible contamination from linear training.	- An aerobic exercise program with music and nonlinear pedagogy reduced anxiety and depression, and improved motor and cognitive functions in boys with ADHD.<br><br>- This approach may be beneficial in educational, clinical, and rehabilitation settings.<br><br>- Future studies should compare linear vs. nonlinear training methods under controlled conditions and include both genders.

Table S1. Characteristic of studies included (continued)

Reference	Objective (AF and EF)	Sample and Age (range)	Study design	Evaluation method	Main findings (effect of PA on EF)	Limitations	Conclusions
Nejati (2021).	- Evaluate the effect of balance-cognitive training (BARAN) on EF in children with ADHD.	Total: 29 boys and girls.<br><br>EG: 15.<br><br>CG: 14.<br><br>Iran.<br><br>7 to 12 years.	Randomized controlled trial with a parallel-group design.<br><br>Participants were randomly assigned to the BARAN EG or the active CG that performed aerobic exercise.	- Cognition tasks: N-Back, Wisconsin Card Sorting Task, Go/No-Go task.<br><br>- Dual-task: Balance and cognitive tasks in a dual-task paradigm.	- BARAN group showed significant improvements in working memory ($p < .001$), cognitive flexibility ($p < .001$), and inhibitory control ($p < .05$).<br><br>- Active CG showed improvements but less pronounced than the BARAN group.	- PA levels between groups were not matched.<br><br>- Subjective physical overload was not measured.<br><br>- The study did not evaluate participants' balance abilities, which could have added insights.	The BARAN intervention, which combines balance and cognitive tasks, was more effective than aerobic exercise at improving EF and ADHD symptoms in children. The dual-task nature of the intervention supports its beneficial effects.
Nejati & Derakhshan (2021).	- Compare the effect of PA with and without cognitive demand on EF and behavioural symptoms in children with ADHD.<br><br>- Evaluate the effect of EXCIR (Exercise for Cognitive Improvement and Rehabilitation) on executive function improvement and symptom amelioration.	Total: 30 boys and girls (mostly male) with ADHD.<br><br>EG1 (EXCIR group): 5 inattentive, 2 hyperactive, 8 combined.<br><br>CG: 6 inattentive, 1 hyperactive, 8 combined.<br><br>Iran.<br><br>9.43 ± 1.43 years.	Randomized, controlled, parallel-group study with repeated measures. Participants were randomly assigned to either:<br><br>- EG1 (EXCIR group): 10–12 sessions of cognitively demanding physical activities (40–50 min/session, 3 sessions/week over 4–5 weeks)<br><br>- Active CG: 10–12 sessions of aerobic running exercise (matched in duration and frequency)	- Cognition tasks: 1-Back Task (working memory: accuracy and response time), Wisconsin Card Sorting Task (cognitive flexibility: number of clusters, perseveration errors, correct responses), Go/No-Go Task (inhibitory control: accuracy on Go/No-Go trials, reaction times).<br><br>- PA task: EXCIR program (12 structured motor-cognitive activities with increasing cognitive demand) and aerobic running exercise (continuous running, no added cognitive load).	The EXCIR group showed significantly greater improvements than the CG in:<br><br>- Working memory: accuracy on the 1-Back task ($p < 0.001$)<br><br>- Cognitive flexibility: increased correct responses and decreased perseveration errors in the WCST ($p < 0.001$)<br><br>- Inhibitory control: improved accuracy in No-Go trials ($p < 0.001$)<br><br>- Behavioural symptoms: significant reduction in ADHD symptoms on Conner's Rating Scale ($p < 0.001$ for parent and teacher ratings)<br><br>These effects were maintained at 1-month follow-up (p values remained < 0.01 for all main outcomes).	- Failure to measure PA load.<br><br>- Short duration between post-test and follow-up.<br><br>- Small sample size and limited female representation.	- PA with cognitive demand (EXCIR) produced stronger and more lasting improvements in EF and behavioural symptoms in children with ADHD than PA without cognitive demand.<br>- Cognitive rehabilitation combining physical and cognitive tasks is a promising non-pharmacological intervention.

Table S1. Characteristic of studies included (continued)

Reference	Objective (AF and EF)	Sample and Age (range)	Study design	Evaluation method	Main findings (effect of PA on EF)	Limitations	Conclusions
Shams et al. (2021).	- Assess the effect of cognitive rehabilitation combined with physical exercise on sustained attention in children with ADHD.<br><br>- Assess the effect on selective and alternating attention.	Total: 40 females with ADHD.<br><br>Yard, Iran.<br><br>9-12 years.<br><br>EG1 (rehabilitation): 10.<br><br>EG2 (physical exercise): 10.<br><br>EG3 (rehabilitation and physical exercise): 10.<br><br>CG: 10.	Randomized, controlled, quasi-experimental clinical trial with pre-test/post-test design.<br><br>Participants were randomly assigned to one of four groups: cognitive rehabilitation (CR), physical exercise (PE), combined CR+PE, and control.<br><br>Interventions delivered via 16 sessions of 60 minutes each over several weeks.	- Cognitive tasks: Sustained attention (IVA-CPT), alternating attention (WCST), selective attention (Stroop test), intelligence (Goodenough-Harris Drawing Test).<br><br>- PA task: Progressive physical exercise program.	- Sustained attention (IVA-CPT): Omission responses significantly lower in the combined group compared to control ($p < .05$).<br><br>- Alternating attention (WCST): Errors significantly lower in combined group vs. control ($p < .05$). Number of correct responses higher in combined and PE groups vs. control ($p < .05$).<br><br>- Selective attention (Stroop test): Interference score and time significantly lower in combined, PE, and CR groups vs. control ($p < .05$).	Not reported by the authors.	Cognitive rehabilitation combined with physical exercise significantly improved sustained, selective, and alternating attention in girls with ADHD, especially in the combined intervention group. Findings suggest synergistic benefits when combining cognitive and physical training.
Suárez-Manzano et al. (2021).	- Evaluate the chronic effects of a 10-week C-HIIT program on selective attention in youth diagnosed with ADHD.	Total: 52 boys and girls.<br><br>EG: 28.<br><br>CG: 24.<br><br>Spain.<br><br>6-16 years with a mean age 10.13 ± 2.68 years.	Quasi-experimental pre-post design.<br><br>EG: Participated in a 10-week C-HIIT program, 2 sessions per week (each 30 minutes), with 16 minutes of high-intensity interval training at 85-100% max heart rate.<br><br>CG: Did not participate in the C-HIIT program.	- Cognitive task: Selective attention (d2 test).<br><br>- PA task: Cooperative High-Intensity Interval Training (C-HIIT).	- Significant improvement in selective attention in the C-HIIT group (pre: 42 ± 30.21 vs. post: 64.21 ± 30.57; $p< 0.001$) with a 21.16% improvement from baseline.<br><br>- Trend towards significant improvement in variation index/stability in the C-HIIT group compared to control ($p= 0.074$), a 16.45% increase.	- Small sample size that may not be representative of the population.<br><br>- Lack of analysis according to ADHD subtypes.<br><br>- Difficulty comparing results due to lack of prior research on monitored C-HIIT in this population.	- A 10-week C-HIIT program significantly improved sleep quality and selective attention in young people with ADHD, but effects on concentration and variation index were inconclusive.<br><br>- An extended C-HIIT program can significantly contribute to improving sleep quality and selective attention in young people with ADHD.<br><br>- No negative effects were observed on any of the study variables, and no injuries occurred during the intervention.

Table S1. Characteristic of studies included (continued)

Reference	Objective (AF and EF)	Sample and Age (range)	Study design	Evaluation method	Main findings (effect of PA on EF)	Limitations	Conclusions
Tsai et al. (2021).	- Examine whether the effects of acute aerobic exercise on inhibitory control in children with ADHD are moderated by exercise intensity.<br><br>- Investigate the role of cortical arousal, as measured by EEG, in mediating the effects of exercise intensity on inhibitory control.	Total: 25 boys and girls with ADHD.<br><br>Taiwan.<br><br>7-12 years, mean 10.54 ± 1.17 years.	- Within-subjects crossover design.<br><br>- Each child participated in three conditions of acute treadmill running (low: 30% HRR, moderate: 50–60% HRR, vigorous: 70–80% HRR), in a counterbalanced order, with at least 3 days between sessions.<br><br>- Each session lasted 30 minutes (5-min warm-up, 20-min main exercise, 5-min cool-down).<br><br>- No non-exercise control condition was included.	Cognitive task: Eriksen flanker task (RT and accuracy), P3 ERP components (amplitude and latency).<br><br>PA task: 20 minutes of treadmill running at three intensities.<br><br>Physiological control: EEG (alpha power), heart rate (pre, during, post), RPE.	- Low and moderate intensity PA resulted in shorter RT than vigorous intensity PA.<br><br>- No significant difference in RT between low and moderate intensity PA.<br><br>- P3 interference effect present after low and vigorous PA, but not moderate intensity PA.<br><br>- Cortical arousal (alpha power) increased with intensity, which was sustained after vigorous PA and decreased after low and moderate PA (moderate effect non-significant).<br><br>- Negative correlation between arousal change and incompatible RT after moderate intensity PA.	- No pre-test or non-exercise control condition.<br><br>- Overlap in exercise intensity classification.<br><br>- Limited generalizability to other ADHD subtypes and girls.<br><br>- Medication use may have confounded results (though 24h abstinence was required).	- Children with ADHD may have better inhibitory control following low and moderate intensity aerobic exercise compared to vigorous intensity exercise.<br><br>- This may be due to increased cortical arousal, as measured by decreased alpha power, following low and moderate intensity exercise.<br><br>- However, conclusions should be interpreted with caution due to the lack of a non-exercise control condition in the study.<br><br>- Findings provide preliminary evidence regarding the optimal intensity of acute exercise for improving inhibitory control in children with ADHD, which is relevant to current PA guidelines.
Wexler et al. (2021).	- Determine whether children show greater reductions in ADHD symptoms after participating in a cognitive training program compared to treatment-as-usual (TAU).<br><br>- Assess whether improvements in targeted cognitive functions (focused attention, response inhibition, working memory) are related to clinical response.	Total: 93 boys and girls with ADHD.<br><br>United States.<br><br>5-9 years with a mean age of 7.3 ± 1.1 years.	Randomized crossover clinical trial.<br><br>Two 15-week periods: one with cognitive training (CT), the other with TAU; 66 children completed both phases.<br><br>CT intervention included: 3–4 weekly 45-minute after-school sessions (30 min of actual computer exercises) of computerized cognitive training (80–150 levels) targeting focused attention, inhibition, and working memory, cognition-enriched physical exercises (45 min/session), and group-based " Good Behaviour Game" to encourage classroom behaviour.	- Cognition task: Flanker Test (focused attention): accuracy and reaction time on incongruent trials, Go/No-Go Test (response inhibition): accuracy on No-Go trials, Working Memory Test: total correct responses.<br><br>- PA task: Cognition-enriched physical exercises.	- 41% of children showed ≥30% reduction in ADHD symptoms after CT vs. 20% during TAU ($p = .02$).<br><br>- Responders to CT showed significantly greater improvements in working memory ($p = .03$), attention ($p = .06$), and inhibition ($p = .09$).<br><br>Improvements in cognitive test performance significantly predicted reductions in ADHD symptoms ($p < .008$).	- Multi-component intervention prevents isolation of effects from specific components.<br><br>- No active control condition and so it is unclear whether the after-school structure contributed to effects.<br><br>- Teacher ratings did not show significant change, raising concerns about real-world generalizability.<br><br>- Symptom improvement, while significant, was modest and similar to typical psychiatric interventions.<br><br>- No data on long-term effects or sustainability of improvements.	- The integrated cognitive training program (computer and physical exercises) reduced ADHD symptoms in a significant proportion of non-medicated children.<br><br>- Improvements in EF (attention, inhibition, working memory) predicted clinical response.<br><br>- Further studies are needed to refine the intervention, examine long-term efficacy, and assess generalizability.

Table S1. Characteristic of studies included (continued)

Reference	Objective (AF and EF)	Sample and Age (range)	Study design	Evaluation method	Main findings (effect of PA on EF)	Limitations	Conclusions
Chang et al. (2022).	Determine if table tennis training can improve EF in children with ADHD.	Total: 48 children with ADHD (grades 1–6; approximately 6–12 years old), both genders.<br><br>EG1: 16.<br><br>EG2: 16.<br><br>CG: 16.<br><br>Taiwan.	Randomized controlled trial.<br><br>- EG1: Received 12 weeks of actual table tennis training, 3 one-hour sessions per week (36 sessions total), including warm-up, main training (with coach or ball-projection machine), gameplay, and cool-down.<br><br>- EG2: Received same duration and frequency of simulated table tennis using Nintendo Wii Sports (exergame).<br><br>- CG: No additional intervention beyond	Cognitive tasks:<br><br>- Stroop Test (selective attention and cognitive inhibition).<br><br>- Wisconsin Card Sorting Test (WCST; cognitive flexibility and problem-solving).<br><br>Physical tasks:<br><br>- None directly assessed in the study (focus was on handwriting performance).	Significant improvement in Stroop Colour-Word score in both EG1 and EG2 vs. CG ($p < .05$), with largest effect in EG2 ($\eta^2 = .170$).<br><br>EG1 showed significant reduction in total errors ($p = .011$, $\eta^2 = .155$) and non-perseverative errors ($p = .003$, $\eta^2 = .213$) vs. CG.<br><br>No significant improvement in EG2 or CG.	- Gender imbalance limits generalizability and precludes gender-based analysis.<br><br>- Restricted age range and so findings are not generalizable to younger/older children.<br><br>- Possibility of practice effects despite inclusion of CG.<br><br>- Lack of long-term follow-up data to assess sustained benefits.<br><br>- No verification of adherence to PA outside of the study.	- Both actual and simulated table tennis training significantly improved handwriting automation and response time in children with ADHD.<br><br>- Only actual table tennis training improved EF as measured by WCST.<br><br>- Findings suggest that concentrative coordination exercises can be effective non-pharmacological strategies to enhance EF and academic-related motor performance in children with ADHD.<br><br>- Further research is recommended to assess long-term effects and generalization to core ADHD symptoms.
de la Merced-García & Jarauta (2022).	- Evaluate the effect of moderate and spontaneous PA, via bike desks, on sustained and selective attention in students with ADHD in a natural classroom setting.	Total: 13 boys and girls with ADHD.<br><br>Spain.<br><br>11-14 years with a mean 12.38 ± 1.19 years.<br><br>EG: 6 (12.67).<br><br>CG: 7 (12.14).	Descriptive, cross-sectional, prospective study with a 14-week intervention using bike desks; groups assigned by convenience.	- Cognitive tasks: d2 Test of Attention (sustained and selective attention), Trail Making Test (cognitive flexibility and attentional control), ADHD-RS-V and Conner' s Teacher Rating Scale (for behaviour and attention ratings).<br><br>- PA task: Daily classroom use of pedal desks during English and Mathematics lessons.	- No statistically significant improvement in sustained or selective attention was observed in the EG compared to the CG after the 14-week intervention.<br><br>- Cognitive test scores did not significantly differ pre- to post-intervention.	- Small sample size (n = 13) and non-random group assignment reduced statistical power.<br><br>- Observation protocols were limited in capturing objective attention data due to classroom dynamics.<br><br>- Possible influence of affective or motivational factors not accounted for in cognitive tests.	- Although no significant improvements in attention were found, this exploratory study highlights the challenges of measuring cognitive changes from spontaneous PA in real classroom settings.<br><br>- Findings underscore the need for larger, controlled studies and for incorporating affective and contextual variables when evaluating the cognitive impact of active classroom interventions like bike desks.

Table S1. Characteristic of studies included (continued)

Reference	Objective (AF and EF)	Sample and Age (range)	Study design	Evaluation method	Main findings (effect of PA on EF)	Limitations	Conclusions
Hattabi et al. (2022).	- Examine the effects of a swimming program on cognitive function, academic performance, and disruptive behaviour in children with ADHD.	Total: 40 with ADHD (mostly male).<br><br>EG: 20.<br><br>CG: 20.<br><br>Tunisia.<br><br>9-12 years.	Cluster-randomized controlled trial.<br><br>- EG: A 12-week recreational swimming program, 3 sessions per week (each 90 minutes), with 15 minutes of warm-up, 70 minutes of aquatic exercises, and 5 minutes of cool-down, led by professional coaches.<br><br>- CG: Did not participate in the swimming program.	- Cognitive task: Inhibitory control (Junior Hayling test).<br><br>- PA task: Recreational swimming program.<br><br>- Physiological control: VO2 max, resting heart rate.<br><br>- Academic performance: Reading comprehension, math, overall grade point average (GPA).	Significant improvement of EG in:<br>- Behaviour ($p < 0.001$), inhibition process ($p < 0.001$), and academic performance ($p < 0.001$).<br><br>- Inhibitory performance, with a 32.34% decrease in part A latency time ($p < 0.001$) and a 41.66% decrease in part B latency time ($p < 0.001$), with large effect sizes.<br><br>- Reading comprehension, Math performance, and overall academic performance (pass marks), with large effect sizes.	- Small sample size.<br><br>- Challenges with sample recruitment and diagnosis.<br><br>- Lack of comparison to typically developing children.<br><br>- Lack of investigation into gender differences.	- A 12-week recreational swimming program positively affected the behavioural, cognitive, and academic performance of children with ADHD.<br><br>- Swimming can be an effective non-pharmacological intervention for improving functioning and academic performance in this population.<br><br>- The swimming intervention protocol used in the study could be adopted by Physical Education teachers and sports medicine professionals as a non-pharmacological treatment for ADHD.
Jiang et al. (2022).	- Assess the way in which aerobic exercise improves executive function in children with ADHD by analysing brain activity using fMRI.<br><br>- Examine changes in brain activity related to the improvement of executive function after the aerobic exercise intervention.	Total: 17 boys and girls with ADHD.<br><br>EG: 17, no CG.<br><br>Changzhou (China).	Pre-post, within-subject, non-controlled design.<br><br>8 weeks of moderate-intensity aerobic exercise (60-69% of maximum heart rate), with 30-minute sessions, 3 times a week, performing rope skipping under parental supervision.	Cognitive Tasks:<br><br>- Flanker task: Used to measure executive function, focusing on response time.<br><br>Physiological Control:<br><br>- fMRI: Changes in brain activity were analysed using two specific measures:<br><br>- Regional Homogeneity (ReHo) in the left middle frontal gyrus and right superior frontal gyrus.<br><br>- Degree Centrality (DC) in the right posterior cingulate cortex.	- ReHo: Significant increase in ReHo values in the left middle frontal gyrus and right superior frontal gyrus after the intervention ($p < 0.001$ before correction, $p < 0.05$ after AlphaSim correction).<br><br>- DC: A significant increase in DC values in the right posterior cingulate cortex was observed after the exercise intervention ($p < 0.05$).<br><br>- Flanker task: Response time on the Flanker task decreased significantly, showing an improvement in executive function ($p < 0.05$).	- Small sample size (17 participants).<br><br>- Poor participant compliance.<br><br>- Difficulties in collecting pre- and post-intervention fMRI data.<br><br>- No CG, which limits ability to compare the effects of the intervention with a non-treated group.<br><br>- Further studies with a larger sample size and additional analysis techniques are needed.	- Moderate-intensity aerobic exercise improves executive function in children with ADHD, specifically increasing brain activity in key areas such as the left middle frontal gyrus, right superior frontal gyrus, and right posterior cingulate cortex.<br><br>- Improvement in executive function was evidenced by a significant decrease in response time on the Flanker task ($p < 0.05$).<br><br>- Findings suggest that aerobic exercise may be a beneficial non-pharmacological intervention for ADHD, but further research is needed to confirm these findings.

Table S1. Characteristic of studies included (continued)

Reference	Objective (AF and EF)	Sample and Age (range)	Study design	Evaluation method	Main findings (effect of PA on EF)	Limitations	Conclusions
Liang et al. (2022).	- Examine the effects of a 12-week combined aerobic and neurocognitive exercise intervention (moderate-to-vigorous PA) on EF (inhibitory control, working memory, cognitive flexibility) and sleep quality in children with ADHD, and to determine whether these effects are maintained 12 weeks post-intervention.	Total: 120 boys and girls (mostly male).<br><br>EG: 40 (ADHD).<br><br>CG: 40 (ADHD wait-list).<br><br>HC: 40 (typical development, age-matched).<br><br>China.<br><br>6-12 years old.	Randomized controlled trial with parallel groups.<br><br>- EG underwent 12-week combined aerobic and neurocognitive training (3 sessions/week, 60 min/session).<br><br>- CG remained on a waitlist during the intervention phase.<br><br>- HC group completed all assessments for comparison.	- Cognition tasks: Arrow Flanker Task (IC), Tower of London (WM), Trail Making Test (CF).<br><br>- PA task: Combined aerobic (e.g., rope skipping, cardio kickboxing, agility ladder) and neurocognitive exercises (e.g., basketball, table tennis, badminton).	EG showed significant improvements in:<br><br>- Inhibitory control (IC): Reduced RT on incongruent Arrow Flanker trials ($p < .05$)<br><br>- Working Memory (WM): Reduced total move scores on Tower of London ($p < .05$)<br><br>- Cognitive Flexibility (CF): Faster completion on Trail Making Test A and B ($p < .05$)	- No pre-intervention control for ball skill level.<br><br>- Over representation of males (77.5%), limiting sex comparisons.<br><br>- High dropout rate (20.5%) in CG.<br><br>- Study was not pre-registered.	- The combined aerobic and neurocognitive exercise program significantly improved EF in children with ADHD, with effects sustained 12 weeks post-intervention.<br><br>- Post-intervention performance approached levels observed in healthy controls.
Nejatifar et al. (2022).	- Evaluate the effectiveness of Dohsa-Hou rehabilitation for response inhibition in children with ADHD.<br><br>- Evaluate the effectiveness of Dohsa-Hou rehabilitation on sustained attention in children with ADHD.	Total: 30 boys.<br><br>EG: 15.<br>CG: 15.<br><br>8-12 years.<br><br>Isfahan, Iran.	Quasi-experimental, randomized controlled pre-test/post-test design.<br><br>EG: Received Dohsa-Hou rehabilitation for 8 months, consisting of 8 one-hour sessions (1 session per month). Each session incorporated techniques such as breathing exercises, body movements, and other rehabilitation tasks designed to enhance attention and self-control mechanisms.<br><br>CG: No intervention during the study period.	Cognitive Tasks:<br><br>- Go-No-Go Test (for response inhibition)<br><br>- Conners Continuous Performance Test (CPT) (for sustained attention)	Response Inhibition:<br><br>The EG showed a significant improvement in response inhibition as measured by the Go-No-Go Test compared to the CG ($p < 0.05$).<br><br>The EG showed a significant improvement in sustained attention as measured by the Conners Continuous Performance Test ($p < 0.05$).	The paper does not mention specific limitations, but a potential limitation could be the lack of a larger, more diverse sample size to generalize results.	- The study demonstrated that Dohsa-Hou rehabilitation has a significant positive effect on improving both response inhibition and sustained attention in children with ADHD.<br><br>- This intervention can be used as a complementary therapeutic approach alongside other psychological treatments for children with ADHD.

Table S1. Characteristic of studies included (continued)

Reference	Objective (AF and EF)	Sample and Age (range)	Study design	Evaluation method	Main findings (effect of PA on EF)	Limitations	Conclusions
Suárez-Manzano et al. (2022).	- Evaluate the chronic effects of a 10-week C-HIIT (Cooperative High-Intensity Interval Training) program on inhibitory control in children and adolescents with ADHD.	Total: 52 boys and girls with ADHD (mostly male).<br><br>EG: 28.<br><br>CG: 24.<br><br>Spain.<br><br>Mean age: 10.13 years.	- Longitudinal intervention study with two groups.<br><br>- 10-week C-HIIT intervention, two 30-minute sessions per week, 6 min warm-up, 16 min of monitored C-HIIT in pairs/groups (≥85% HRmax), 8 min cool-down.	- Cognitive tasks: Stroop Test (Interference, Inhibition).<br><br>- PA task: Cooperative High-Intensity Interval Training (C-HIIT) protocol.<br><br>- Physiological control: Heart rate monitors used to confirm ≥85% HRmax.	- Inhibitory control: Interference: +11% improvement in C-HIIT group vs. control ($p = 0.002$).<br><br>- Inhibition: +8% improvement in C-HIIT group ($p < 0.041$).	- Further research is needed to determine the most appropriate exercises based on the age, sex, and motivations of participants. Findings should be interpreted with caution.<br><br>- Results may lack generalizability.	- A 10-week C-HIIT program significantly improved inhibitory control in children and adolescents with ADHD.<br><br>- The program was effective even with only two sessions per week, making it practical for school settings.<br><br>- Authors suggest integrating C-HIIT into the school day (e.g., recess or breaks) to promote cognitive and behavioural benefits without reducing academic time.
Ji et al. (2023).	- Investigate the effect of exergaming on attention in children with ADHD.<br><br>- Compare the effects of exergaming and aerobic exercise (bicycle exercise) on attention in children with ADHD.	Total: 42 children with mild to moderate ADHD (mostly male).<br><br>Final sample after dropout: 30 children.<br><br>EG1 (exergaming group): 16.<br>EG2 (bicycle exercise group): 16.<br><br>8–12 years.<br><br>South Korea.	Randomized controlled trial (RCT) without CG.<br><br>EG1: Played the "Alchemist's Treasure" game using the ExerHeart device, 3 sessions per week, 50 minutes per session, at 60–80% heart rate reserve, for 4 weeks.<br><br>EG2: Performed stationary cycling exercise under the same frequency, duration, and intensity conditions.<br>Both interventions lasted 4 weeks. The study was double-blinded in terms of assessment and data analysis.	Cognitive tasks:<br><br>- Go/No-go task: measured response time (RT) and accuracy.<br><br>- FAIR test (Frankfurter Aufmerksamkeits-Inventar): measures selective attention (P), self-control (Q), and persistent attention (C).<br><br>- EEG recording: assessed event-related potentials (ERP), specifically N2 amplitude and latency.<br><br>PA task: Not applicable beyond the structured interventions.<br><br>Physiological control: Exercise intensity was controlled by monitoring heart rate reserve (target: 60–80%).	- Go/No-go task: Both groups showed reduced RTs post-intervention (interaction effect: $F(1,28)=8.03$, $p=.008$, $\eta^2_p=0.223$).<br><br>- ERP (N2 amplitude): Greater increase in N2 amplitude in EG1 than EG2 ($F(1,28)=6.76$, $p=.015$, $\eta^2_p=0.195$), indicating enhanced attentional control.<br><br>- FAIR test: Both groups showed significant improvements in:<br><br>Selective attention (P): $F(1,28)=14.37$, $p<.001$, $\eta^2_p=0.339$<br><br>Self-control (Q): $F(1,28)=7.65$, $p=.010$, $\eta^2_p=0.215$<br><br>Persistent attention (C): $F(1,28)=6.82$, $p=.014$, $\eta^2_p=0.196$.<br><br>No significant differences between groups in behavioural scores, but EG1 had larger neurophysiological gains.	- No non-exercise CG to isolate the effect of PA.<br><br>- Small sample size and homogeneous population limit generalizability.<br><br>- Only one exergame used, limiting conclusions about exergaming more broadly.<br><br>- Game performance data were not analysed in relation to attention outcomes.	Exergaming and bicycle exercise both improved attention-related EF in children with ADHD. However, exergaming showed greater neurophysiological effects, suggesting it may be a more engaging and cognitively stimulating alternative for ADHD interventions. Further research is needed to confirm these results and explore long-term effects.

Table S1. Characteristic of studies included (continued)

Reference	Objective (AF and EF)	Sample and Age (range)	Study design	Evaluation method	Main findings (effect of PA on EF)	Limitations	Conclusions
Jun (2023).	Explore the impact of soccer practice on executive function in boys with ADHD.	Total: 968 (boys).<br><br>6-8 years.<br><br>China.	Randomized, parallel-group, observational study.<br><br>EG: Received a 6-week soccer intervention, in addition to regular school sports activities.<br><br>CG: Only participated in regular school sports activities.	- Cognitive task: E-Prime 2.0 (inhibition control, working memory, cognitive flexibility).<br><br>- PA task: Soccer practice intervention for the EG (6 weeks, integrated into regular school sports activities).	- Inhibition control: Significant improvement from pre-test to post-test ($p < 0.01$).<br><br>- Cognitive flexibility: Significant improvement over time compared to control and conventional PE groups ($p < 0.01$).<br><br>- Working memory: No significant difference between soccer intervention and conventional PE groups.	- Limited sample size.<br><br>- Did not explore effects on different ADHD subtypes, ages, or genders.<br><br>- Did not compare effects of different exercise intensities.<br><br>- Some unexpected findings that were not fully explained.	- A 6-week soccer practice intervention can improve executive function, especially inhibition control and cognitive flexibility, in 6-8-year-old boys with ADHD, but does not have a significant effect on memory.<br><br>- Positive effects of both soccer and conventional Physical Education activities on the three sub-functions of executive function (inhibition control, working memory, and cognitive flexibility) increase over time.<br><br>- The soccer activity group showed greater improvement in inhibition control and cognitive flexibility compared to the conventional Physical Education group, but the two groups did not differ significantly in terms of impact on working memory.
Liang (2023).	Examine the effects of PA interventions on EF in children with ADHD.	Total: 120 (mostly male).<br><br>ADHD EG: 40.<br><br>ADHD CG: 40.<br><br>Typical CG: 40.<br><br>China.<br><br>6-12 years.<br><br>EG: 8.37 ± 1.42.<br><br>ADHD CG: 8.29 ± 1.27.<br><br>Health CG: 8.49 ± 1.51.	Study 3: Randomized controlled trial examining the effects of a 12-week combined aerobic and cognitive-engaging PA intervention on EF in children with ADHD, compared to a waitlist CG and a healthy CG.<br><br>80 children with ADHD, randomly assigned to either a PA EG or a waitlist CG.	-Cognitive tasks: Neurocognitive task.<br><br>-PA: 12-week PA program consisting of 36 sessions (3 sessions per week, 60 minutes per session). Each session included a warm-up (10 minutes), aerobic exercise (20 minutes), cognitive-engaging exercise (20 minutes), and a cool-down (10 minutes). Exercise intensity was kept at 60-80% of maximum heart rate.<br><br>-Physiological monitoring: HR.	- PA interventions show beneficial effects on overall EF in children with ADHD, with both aerobic and cognitive-engaging exercise having a similar positive impact.<br>- A 12-week combined PA intervention (aerobic and cognitive-engaging exercise) improved EF in children with ADHD, and effects were sustained for 12 weeks after the intervention.	- Limited sample size.<br><br>- Did not explore effects on different ADHD subtypes, ages, or genders.<br><br>- Did not compare effects of different exercise intensities.<br><br>- Some unexpected findings that were not fully explained.<br>- No control over other treatments.<br><br>- Unclear causality between sleep and executive function.<br><br>- Results limited to specific age and ADHD group.<br><br>- No long-term follow-up.<br><br>- CG lacked active intervention.	- PA interventions are beneficial non-pharmacological treatments that can improve EF and sleep quality in children with ADHD.<br>- A 12-week PA program has lasting benefits for children with ADHD, and effects are sustained for at least 12 weeks after the intervention.

Table S1. Characteristic of studies included (continued)

Reference	Objective (AF and EF)	Sample and Age (range)	Study design	Evaluation method	Main findings (effect of PA on EF)	Limitations	Conclusions
Ludyga (2023).	- Examine the effects of judo training on behavioural and neurocognitive indices of response inhibition in children born very preterm and children with ADHD.<br><br>- Compare the treatment effects of judo training between the two study groups (children born very preterm and children with ADHD) and confirm whether benefits generalize across different conditions with cognitive control deficits.	Total: 113 children (mostly male).<br><br>EG1 (CHIPMANC): 28<br><br>CG1: 28.<br><br>8-14 years.<br><br>EG2 (JETPAC): 56.<br><br>CG2: 28.<br><br>8-12 years.<br><br>Switzerland and Germany.	Randomized, double-blind, parallel, multi-site controlled trial.<br><br>EG: The judo intervention consisted of 60-minute sessions twice a week for 12 weeks, focusing on learning basic judo techniques, physical fitness, and judo-specific sparring (Randori).<br><br>CG: Waitlist.	Cognitive tasks:<br><br>- Computerized Go/NoGo task to assess response inhibition (reaction time, omission error rate, commission error rate).<br><br>- Event-related potentials (ERPs) from the Go/NoGo task (N2, P3a, P3b).<br><br>Physical tasks:<br><br>- Submaximal cycling ergometer test (PWC170).<br><br>- Movement Assessment Battery for Children-2 (MABC-2) for motor skills.	Children born very preterm (CHIPMANC):<br><br>- Judo training reduced the commission error rate on the Go/No-Go task ($p < 0.05$) and increased P3a amplitude ($p < 0.01$) compared to the CG.<br><br>Children with ADHD (JETPAC):<br><br>- Judo training did not show significant effects on commission error rate or ERP components.	- Slight differences in inclusion criteria and randomization between the two studies.<br><br>- Inability to detect effects on aerobic fitness and motor skills, leaving the mechanism unclear.<br><br>- Lack of monitoring for other activities that could improve response inhibition.<br><br>- Use of an abstract cognitive task rather than more ecologically valid assessments.<br><br>- Inability to fully explain cognitive benefits through the measured ERP components.<br><br>- Inability to generalize findings beyond the specific conditions studied (very preterm birth and ADHD).	- Judo training improved response inhibition and increased P3a amplitude in children born very preterm, but not in children with ADHD.<br><br>- The benefits observed in children born very preterm were achieved without changes in motor skills or aerobic fitness, suggesting that effects were specific to the cognitive demands of judo.<br><br>- Other cognitive control processes, such as conflict monitoring and attentional resource allocation, were not affected by judo training.
Barudin-Carreiro et al. (2024).	- Evaluate the feasibility of conducting a rigorous experimental study comparing the effects of walking and standing on EF in children with ADHD.	Total: 22 children with DHD.<br>6-11 years.<br><br>United States.	Randomized, parallel-group pilot study with three experimental conditions.<br><br>EG 1 (Walking): 20 min.<br><br>EG 2: (Standing): 20 min.<br><br>EG 3: (Sitting) 20 min.<br><br>Participants listened to an age-appropriate music playlist during the intervention whilst their heart rate was monitored.	Cognitive task:<br><br>- Stroop Colour-Word Test (SCWT) to measure inhibition/inhibitory control.<br><br>- Wisconsin Card Sorting Task (WCST) to measure problem solving, cognitive flexibility, planning, updating, and impulsive responding.<br><br>Physiological control: Heart rate, height, weight, BMI.	Stroop Colour-Word Test:<br><br>- Sitting produced the largest improvement in inhibition ($p < 0.05$).<br><br>- Walking led to less improvement than standing and sitting.<br><br>Wisconsin Card Sorting Test (WCST):<br><br>- Standing brought about the largest improvement in all categories ($p < 0.05$).<br><br>- Walking and sitting led to moderate improvements, with standing being the most effective.	- Small sample size, increasing the risk of Type II errors.<br><br>- Performance and detection bias due to a single researcher conducting the study.<br><br>- Testing effects and fatigue from repeated assessments.<br><br>- Early termination due to the COVID-19 pandemic, which reduced the sample size.	- The study design was feasible for examining the effects of standing on executive function in children with ADHD, and larger scale studies should be conducted.<br><br>- Standing did not significantly improve executive function, but trends were consistent with previous research showing that PA improves executive function in children.<br><br>- Further research could have implications for school systems and treatment options for ADHD.

Table S1. Characteristic of studies included (continued)

Reference	Objective (AF and EF)	Sample and Age (range)	Study design	Evaluation method	Main findings (effect of PA on EF)	Limitations	Conclusions
Sun et al. (2024).	- Examine the effectiveness of an 8-week game-based HIIT (GameHIIT) program on executive function and cerebral hemodynamic responses in children with ADHD, compared to a game-based structured aerobic exercise program (GameSAE) and a CG.	Total: 49 boys and girls with ADHD (mostly male).<br><br>GameHIIT: 16.<br><br>GameSAE: 15.<br><br>CG: 18.<br><br>Hong Kong.<br><br>8-13 years with a mean age of 10.10 ± 1.83 years.	- Three-arm, partially-blinded randomized controlled trial.<br><br>- Participants randomly assigned to GameHIIT, GameSAE, or control.<br><br>GameHIIT: 8 weeks. 2 sessions/week, ~30 min each (rugby-based HIIT games).<br><br>GameSAE: 8 weeks. Up to 2 sessions/week, ~60 min (aerobic games).<br><br>CG: Maintained regular activity.	- Cognitive tasks: (1) Wisconsin Card Sorting Test (WCST) for flexibility cognition, (2) Colour-Word Stroop Test (CWST) for control inhibition, (3) Tower of London Test (ToLT) for planning and organization, (4) Corsi Block-Tapping Test (CBTT) for working memory, (5) Behaviour Rating Inventory of Executive Function (BRIEF-II, parent-report).<br><br>- PA tasks: ALPHA fitness battery: handgrip strength, long jump, shuttle run, beep test.<br><br>- Physiological control: fNIRS (functional near-infrared spectroscopy) for cerebral haemodynamics, accelerometers and PAQ-C for PA.	- No significant improvement in EF (performance-based or parent-reported), except for improved self-monitoring in the GameSAE group.<br><br>- Cerebral haemodynamic response showed some activation changes, but inconclusive.	- Small sample size and high dropout rate due to COVID-19.<br><br>- Short duration and no follow-up.<br><br>- Parent-reported data may introduce bias.<br><br>- Medication use not clearly reported.<br><br>- Energy expenditure not directly measured.	- Game-based HIIT and aerobic exercise improved PA and fitness in children with ADHD.<br><br>- No consistent improvements in executive function or ADHD symptoms.<br><br>Future studies should optimize intervention design and duration, and include long-term follow-up.
Huang et al. (2024).	- Investigate the effects of an 8-week rope skipping exercise (RSE) intervention on working memory (WM) in children with ADHD.	Total: 88 boys and girls.<br><br>55 with ADHD and 27 typically developing.<br><br>EG1 (AWRSE): 22 with a mean age of 10.18 ± 1.10 years.<br><br>EG2 (AWSG): 33 with a mean age of 9.38 ± 0.96 years.<br><br>CG: 27 with a mean age of 8.94 ± 0.56 years.<br><br>China.<br><br>6-12 years.	Randomized, double-blind, parallel-group controlled trial.<br><br>EG1 (AWRSE): rope skipping exercise.<br><br>EG2 (AWSG): sports games.<br><br>CG: sports games (typically developing children).<br><br>8 weeks. 2 sessions/week. 60 minutes. 16 sessions.	- Cognitive tasks: Working memory assessed with 1-back and 2-back tasks (response time and accuracy).<br><br>- PA task: rope skipping exercise or sports games.<br><br>- Physiological control: Heart rate monitored using Polar sensors to confirm intensity.	Working memory (WM):<br><br>- AWRSE group showed significant improvements post-intervention in accuracy and response time on the n-back task ($p = .011$).<br><br>- No significant WM improvement in AWSG or CG.	- Small sample size.<br><br>- ADHD children did not show expected baseline WM deficits compared to TD group.<br><br>- No analysis by gender.<br><br>- ADHD subtypes not considered separately.	- An 8-week rope skipping exercise significantly improved working memory in children with ADHD.<br><br>- Sports game intervention did not produce the same benefits.<br><br>- RSE appears to be an effective and accessible intervention for children with ADHD.

Table S1. Characteristic of studies included (continued)

Reference	Objective (AF and EF)	Sample and Age (range)	Study design	Evaluation method	Main findings (effect of PA on EF)	Limitations	Conclusions
Zhao et al. (2024).	- Evaluate the efficacy of the BrainFit digital intervention at reducing ADHD symptoms in school-aged children with ADHD.<br>- Evaluate efficacy of the BrainFit digital intervention for improving executive function in school-aged children with ADHD.	Total: 90 (mostly male).<br><br>EG: 44.<br><br>CG: 46.<br><br>China.	A randomized, controlled trial design.<br><br>EG: received the BrainFit digital cognitive-physical intervention. This intervention consisted of 12 sessions over 4 weeks, with 3 sessions per week (Monday, Wednesday, and Friday). Each session lasted 30 minutes and was delivered via iPad under supervision. The intervention combined cognitive and physical tasks, targeting both executive function and physical skills. The program included 6 adaptive modules where participants	Cognitive task:<br><br>BRIEF parent form, including the Global Executive Composite score, Behavioural Regulation Index, Metacognition Index, and their respective clinical scales.	- Significant improvements in executive function, specifically in the metacognition and global executive function domains (Metacognition Index: t = 3.1, p = 0.004, Global Executive Composite: t = 2.9, p = 0.006).<br><br>- Smaller improvements in the behavioural regulation domain (t = 1.9, p = 0.058).	- Small sample size, limiting the ability to analyse results in detail.<br><br>- Lack of control for ADHD medication use, ADHD subtype, and age distribution, limiting generalizability.<br><br>- Only measured immediate outcomes, unable to assess durability of effects.<br><br>- Lack of blinding and placebo control, which could have introduced bias.	- The study concludes that the BrainFit digital intervention, which combines cognitive training and exercise, was effective at improving ADHD symptoms and executive function in children with ADHD compared to a CG.<br><br>- The intervention has the potential to increase access to ADHD treatment, especially in areas with limited resources.

Note: Physical activity (PA), executive function (EF), experimental group (EG), control group (CG).
